# Hippocampal Atrophy as a Quantitative Trait in a Genome-Wide Association Study Identifying Novel Susceptibility Genes for Alzheimer's Disease

**DOI:** 10.1371/journal.pone.0006501

**Published:** 2009-08-07

**Authors:** Steven G. Potkin, Guia Guffanti, Anita Lakatos, Jessica A. Turner, Frithjof Kruggel, James H. Fallon, Andrew J. Saykin, Alessandro Orro, Sara Lupoli, Erika Salvi, Michael Weiner, Fabio Macciardi, for the Alzheimer's Disease Neuroimaging Initiative

**Affiliations:** 1 Department of Psychiatry and Human Behavior, University of California Irvine, Irvine, California, United States of America; 2 Department of Sciences and Biomedical Technologies, University of Milan, Segrate (MI), Italy; 3 Department of Biomedical Engineering, University of California Irvine, Irvine, California, United States of America; 4 Center for Neuroimaging, Department of Radiology and Imaging Sciences, Indiana University School of Medicine, Indianapolis, Indiana, United States of America; 5 Istituto di Tecnologie Biomediche, Consiglio Nazionale delle Ricerche, Segrate (MI), Italy; 6 Universita' Vita e Salute, Department of Neurology and Neuroscience, Milan, Italy; 7 Department of Radiology, Psychiatry and Neurology, University of California San Francisco, San Francisco, California, United States of America; University of Muenster, Germany

## Abstract

**Background:**

With the exception of APOE ε4 allele, the common genetic risk factors for sporadic Alzheimer's Disease (AD) are unknown.

**Methods and Findings:**

We completed a genome-wide association study on 381 participants in the ADNI (Alzheimer's Disease Neuroimaging Initiative) study. Samples were genotyped using the Illumina Human610-Quad BeadChip. 516,645 unique Single Nucleotide Polymorphisms (SNPs) were included in the analysis following quality control measures. The genotype data and raw genetic data are freely available for download (LONI, http://www.loni.ucla.edu/ADNI/Data/). Two analyses were completed: a standard case-control analysis, and a novel approach using hippocampal atrophy measured on MRI as an objectively defined, quantitative phenotype. A General Linear Model was applied to identify SNPs for which there was an interaction between the genotype and diagnosis on the quantitative trait. The case-control analysis identified APOE and a new risk gene, TOMM40 (translocase of outer mitochondrial membrane 40), at a genome-wide significance level of≤10^−6^ (10^−11^ for a haplotype). TOMM40 risk alleles were approximately twice as frequent in AD subjects as controls. The quantitative trait analysis identified 21 genes or chromosomal areas with at least one SNP with a p-value≤10^−6^, which can be considered potential “new” candidate loci to explore in the etiology of sporadic AD. These candidates included EFNA5, CAND1, MAGI2, ARSB, and PRUNE2, genes involved in the regulation of protein degradation, apoptosis, neuronal loss and neurodevelopment. Thus, we identified common genetic variants associated with the increased risk of developing AD in the ADNI cohort, and present publicly available genome-wide data. Supportive evidence based on case-control studies and biological plausibility by gene annotation is provided. Currently no available sample with both imaging and genetic data is available for replication.

**Conclusions:**

Using hippocampal atrophy as a quantitative phenotype in a genome-wide scan, we have identified candidate risk genes for sporadic Alzheimer's disease that merit further investigation.

## Introduction

Age is a major risk factor for Alzheimer's disease (AD), and with both life expectancy and population increases, its incidence is expected to double in the next two decades [1]. Genetic investigations have discovered some putative pathophysiological mechanisms related to the accumulation of senile plaques and neurofibrillary tangles, the histopathological hallmarks of AD. The high incidence of dementia in Down's syndrome and analysis of the amino acid sequence of beta-amyloid has led to identification of a series of mutations associated with increased amyloid production. However, such mutations only account for a very small percentage of Alzheimer cases. Risk factors have been identified for the sporadic cases, which are the majority of AD patients, although these factors collectively explain a relatively modest amount of the genetic risk for AD [2]. The most significant of these is the presence of apolipoprotein E4 (APOE ε4). The most commonly used strategies to find disease-related genes such as APOE have been linkage studies and candidate gene association studies. The late onset of Alzheimer's disease and the sporadic nature of most cases complicate family studies, although more than a dozen have been performed [2]. These family studies and the case-control studies strongly implicate multiple genetic loci in susceptibility to AD [2]. Candidate gene approaches targeting amyloid, tau, and inflammatory cascades have proved useful, yet the identification of other major risk factors have proved elusive, with few associations being replicated.

The recent availability of high-throughput genotyping has allowed the simultaneous measurement of hundreds of thousands to more than one million SNPs (Single Nucleotide Polymorphisms), which has made genome-wide association studies (GWAS) feasible. A major challenge in case-control GWAS designs in which allele and genotype frequencies are compared between AD and control patients is achieving sufficient statistical power. Such categorical approaches require approximately 6,000 cases and controls to obtain 85% power to detect a 30% difference (odds-ratio of 1.3) with a minor allele frequency of 0.15 [3]. Quantitative trait association studies offer several advantages over case-control studies, since the dependent measures are quantitative and more objective than diagnostic categorization, and can increase the statistical power four to eight fold, thus greatly decreasing the required sample size to achieve sufficient statistical power [4].

We present the results of a GWAS in the Alzheimer's Disease Neuroimaging Initiative (ADNI) cohort, a longitudinal multi-site observational study including AD, mild cognitive impairment (MCI), and elderly individuals with normal cognition [5] assessing clinical and cognitive measures, MRI and PET scans (FDG and ^11^C PIB) and blood and CNS biomarkers. Major goals of ADNI are to make this wealth of data available to the general scientific community and to improve methods for clinical trials by optimizing methods for imaging that are associated with clinical symptoms and the conversion of MCI to AD. The clinical and imaging measures from this longitudinal study have previously been made publicly available; the genome-wide genotyping results for 793 subjects from the Illumina Human610-Quad BeadChip (Illumina Corporation, San Diego, CA) as performed by The Translational Genomics Research Institute (TGen, Phoenix, AZ), are now also publicly available.

In this initial analysis of the AD and the healthy control subsets of the data, we first performed a case control analysis and found APOE as well as an associated gene TOMM40, that provides additional risk for developing AD.

To complement the case-control/candidate gene approach to discover new risk genes for AD we performed a QT (quantitative trait) analysis. Our method uses the hippocampal grey matter density from neuroimaging as the quantitative trait, and examines which SNPs (as proxies for genes) influence the quantitative trait differently for AD and healthy controls. The concentration of grey matter in the hippocampus [6] was chosen as the QT phenotype as it is affected early in AD, is implicated in the conversion to and progress of AD [7], [8] and is associated with many of the cardinal symptoms, and it can be reliably measured *in vivo*. We used differences in the total grey matter concentration of the hippocampal area, (the four CA fields of the hippocampus proper, plus dentate gyrus, subiculum, entorhinal cortex, and parahippocampal gyrus) in AD subjects vs normal aged-matched controls in our model, since MRI studies have suggested that reductions in hippocampus over time can be particularly useful in predicting AD before the onset of clinical symptoms, and in assessing the efficacy of pharmacological treatment in clinical trials [9]–[12]. Therefore, in our GWA study we used hippocampal concentration as an imaging phenotype to reveal genes that potentially influence hippocampal atrophy and dementia in the context of AD. The genes which influence hippocampal grey matter concentration differentially in AD and healthy subjects may provide important information regarding the mechanisms of disease-related atrophy. The power of this approach is demonstrated in the results implicating novel genes which have been implicated in candidate neurodegenerative mechanisms.

## Methods

### Ethics

For the purpose of this analysis we used ADNI subject data that was previously collected across 50 sites. Study subjects gave written informed consent at the time of enrollment for imaging and genetic sample collection and completed questionnaires approved by each participating sites' Intitutional Review Board (IRB).

### ADNI

Data used in the preparation of this article were obtained from the Alzheimer's Disease Neuroimaging Initiative (ADNI) database (www.loni.ucla.edu/ADNI). The ADNI was launched in 2003 by the National Institute on Aging (NIA), the National Institute of Biomedical Imaging and Bioengineering (NIBIB), the Food and Drug Administration (FDA), private pharmaceutical companies and non-profit organizations, as a $60 million, 5-year public-private partnership. The primary goal of ADNI has been to test whether serial magnetic resonance imaging (MRI), positron emission tomography (PET), other biological markers, and clinical and neuropsychological assessment can be combined to measure the progression of mild cognitive impairment (MCI) and early Alzheimer's disease (AD). Determination of sensitive and specific markers of very early AD progression is intended to aid researchers and clinicians to develop new treatments and monitor their effectiveness, as well as lessen the time and cost of clinical trials.

The Principle Investigator of this initiative is Michael W. Weiner, M.D., VA Medical Center and University of California – San Francisco. ADNI is the result of efforts of many co-investigators from a broad range of academic institutions and private corporations, and subjects have been recruited from over 50 sites across the U.S. and Canada. The initial goal of ADNI was to recruit 800 adults, ages 55 to 90, to participate in the research—approximately 200 cognitively normal older individuals to be followed for 3 years, 400 people with MCI to be followed for 3 years, and 200 people with early AD to be followed for 2 years.” For up-to-date information see www.adni-info.org.

### Participants

All subjects were part of the Alzheimer's disease Neuroimaging Initiative (ADNI), a longitudinal multi-site observational study including AD, mild cognitive impairment (MCI), and elderly individuals with normal cognition assessing clinical and cognitive measures, MRI and PET scans (FDG and ^11^C PIB) and blood and CNS biomarkers. Brain imaging, biological samples, and clinical assessments are longitudinally collected for a target of 200 healthy controls, 400 MCI, and 200 AD subjects. For the AD sample, the study focuses on identification of sporadic cases of mild AD, subjects between the ages of 55–90, with an MMSE score of 20–26 inclusive and meeting NINCDS/ADRDA criteria for probable AD [13]–[15], and having an MRI consistent with the diagnosis of AD. Major goals of ADNI are to make this wealth of data available to the general scientific community and to improve methods for clinical trials by optimizing methods for imaging that are associated with diagnosis and clinical symptoms. In this analysis we contrast the AD subjects with healthy controls. This analysis is confined to baseline diagnosis and MRI scans for each subject.

Following volumetric analysis and genotyping quality control measures (described below), data from a total of 172 AD subjects and 209 healthy controls were included in this analysis.

### Procedures

#### MRI

The data collection methods have been described in more detail in Jack et al., 2008 [16], and Mueller et al. 2005 [5]. The images analyzed here were the baseline or screening scans at the entry to the study; they were collected using the standard ADNI protocol on 1.5T GE, Siemens, or Philips MR scanners [16].

The analyzed datasets included the T1-weighted structural MRI scans from all available subjects. Following the segmentation and quality assurance defined below, a final dataset of 229 healthy controls and 194 AD subjects provided grey matter concentration estimates of the hippocampus, using a voxel-based morphometry approach.

The MRI images were converted from DICOM format into NIFTI format and re-oriented into MNI coordinate conventions using FSL tools [17]. They were then registered with the MNI brain atlas, corrected for intensity inhomogeneities and segmented into 3 classes (grey matter (GM), white matter (WM), and CSF) using the VBM toolbox integrated with the SPM5 package [6], [18].

Datasets with an extreme value of computed total brain density or the ratio of grey/white matter density were inspected, leading to the exclusion of several datasets in which segmentation was unsuccessful. Often, these were cases with large ventricles and/or diffuse white matter hypointensities. When there were multiple datasets per subject, the best segmentation for each subject was selected, blind to diagnosis or gender. The datasets of all subjects whose segmentation was successful were used to construct custom templates for the class prior probabilities. These custom templates were used in a second run to segment all datasets. To allow a comparison with the previous run, the same datasets were selected for further evaluation. Again, a quality check was performed using the total brain density and the ratio of grey/white matter density. The total grey matter was generally higher in this segmentation, which is very much expected with a better-matched template.

The right and left hippocampal regions were defined using the AAL brain atlas [19], and the mean of the grey-matter fraction over voxels within those regions was computed for the final imaging phenotypes on each subject.

#### Genotyping

Blood samples were obtained from each participant and sent to Pfizer for DNA extraction. All DNA extraction and genotyping was done blinded to group assignment. Genotyping on the blinded DNA was performed by TGen using the Illumina Human610-Quad BeadChip.

Approximately 200 ng of DNA was used to genotype each subject sample according to the manufacturer's protocol (Illumina, San Diego, CA). After amplification, fragmentation and hybridization, the specifically hybridized DNA was fluorescently labeled by a single base extension reaction and detected using a BeadArray scanner. Non-specifically hybridized fragments were removed by washing while remaining specifically hybridized DNA were processed for the single base extension reaction, stained and imaged on an Illumina Bead Array Reader. Normalized bead intensity data obtained for each sample were loaded into the Illumina BeadStudio 3.2 software which generated SNP genotypes from fluorescent intensities using the manufacturer's default cluster settings. The data analyzed with BeadStudio are publicly available on the LONI website (www.loni.ucla.edu/ADNI/Data). The raw genotypic data were imported in a genome-wide data management system, i.e. SNPLims [20], to allow the export of user-defined formats compatible with the genetic programs used for the statistical analysis.

The following Quality Control (QC) procedures were implemented on the genome-wide data. We performed all data analysis and QC using the PLINK software package (http://pngu.mgh.harvard.edu/purcell/plink/), release v1.05. DNA samples with a call rate<95% were excluded. The average call rate in the remaining samples was 99.4%. From a total of 600,470 SNPs in autosomal chromosomes (sex chromosomes, mitochondrial, and pseudo-autosomal SNPs were excluded), the intensity-only probes from chromosomes 1–22 were excluded (17,879), and 5,592 SNPs were excluded by the PLINK algorithm. SNPs were included in the imaging genetics analysis if their minor allele frequency (MAF) was>.05, were present for>90% of the subjects, and did not depart from Hardy-Weinberg equilibrium for the controls using a threshold of 10^−3^. (Some SNPs were excluded for more than one category). The final number of SNPs included in the analyses is 516,645.

Since the Illumina chip does not include the SNPs associated with APOE alleles, a separate analysis was performed in which DNA was extracted from 3 ml blood samples. APOE genotyping was carried out on these samples by PCR amplification and HhaI restriction enzyme digestion. The genotype was resolved on 4% Metaphor Gel and visualized by ethidium bromide staining [21].

#### Statistical Analysis

Differences in clinical and demographic values between these groups were analyzed by t test, chi-square or Fisher exact test using STATA10 [22]. Population stratification was examined with PLINK v1.5 and EIGENSTRAT [23], [24] and a lambda of 1.06 was observed, indicating no stratification.

#### Case-control analysis

Because APOE ε4 is a well documented risk factor for late onset AD, the Cochran-Armitage trend test was used to analyze the APOE ε4 allele (which are results of “diplotypes” constructed on SNPs rs429358 and rs7412) distribution between healthy controls and AD patients.

Based on the significance of our finding regarding APOE ε4 in our sample and recent interest in this region we expanded our genotyping in the APOE region (∼300-Kb on chromosome 19). To increase the map density in the APOE region, we 1) merged the results of the genotyping (performed with the APOE-specific genotyping) of SNPs rs429358 and rs7412 and 2) performed imputation. To impute the region, the set “hapmap_r23” was downloaded from the HapMap site. This dataset includes 270 subjects from each one of the 4 populations studied by HapMap, i.e. CEU, YRB and CHB+JPT for 267 SNPs [25]. The imputation was considered successful for 159 SNPs with an in-silico call rate>90% in this region. After imputation, the new dataset was used to calculate case-control association tests: 1) single marker association and 2) haplotypes proxy association. The proxy association test was calculated using the flanking markers in strong LD with the “reference” SNP (i.e. proxies).

#### Quantitative Trait analysis

All autosomal SNPs that passed quality control checks were tested for quantitative trait association interaction using the general linear model as implemented in PLINK v1.5. The statistical model is based on comparing the differential effects of SNP association by diagnosis on the brain imaging quantitative trait, i.e. the interaction term.

Based on the demographic analysis of our sample and the case-control allelic association test for APOE ε4, we covaried for age and gender and for the number of APOE ε4 alleles in the model. Out of the possible 4 models to choose from (i.e. additive, codominant, dominant and recessive) we *a priori* chose the additive model as it is the most general, both reflecting the additive contribution to risks for complex diseases [26], and detecting strong non-additive effects.

The general linear model used to determine the interaction term was:
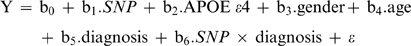
in which b_i_ represents the coefficients, *SNP* represents the genotype of each marker tested, APOE ε4 represents the allele dose of ε4, gender and diagnosis were coded as dichotomous variables, and age is the age of the subject in years.

Our focus is on the SNP×diagnosis interaction. There is no accepted method for determining a statistical threshold for a quantitative trait interaction in a context of a genome-wide association study (GWAS). Given 516,645 SNPs and the sample size of 381 subjects, any result at 10^−6^ or smaller provides statistical evidence for a main effect associating a given SNP with a quantitative trait. There is a lack of consensus on how to identify a genome-wide significance threshold. Reaching a consensus definition for an appropriate statistical threshold for the interaction term in a GWAS is even more complex. For the purposes of presenting the initial analyses of our data and considering the small sample size, the number of variables analyzed and the complexity of the model, we chose a conservative hypothesis for a threshold of 10^−6^ for our interaction term. In keeping with recommendations for presenting a GWAS analysis [27] the results of all analyses are provided in the supplemental material.

The Linkage Disequilibrium (LD) maps of each chromosomal region harboring the most significant SNPs were created using a window size of±200 Kb. D' values were calculated using Haploview 4.1 [28] and visualized with WGAViewer software, Version 1.25W; 2009 (http://www.genome.duke.edu/centers/pg2/downloads/wgaviewer.php) [29]. The genetic annotation was performed using WGAViewer: SNPs location and gene names were derived from Ensembl release 52 - Dec 2008 (http://www.ensembl.org/) [30].

## Results

Table 1 represents the demographic characteristics of the sample analyzed. In this population the healthy control sample had significantly more education than the AD sample, as well as the expected differences in measures of cognitive performance.

**Table 1:** Baseline characteristic of mild AD subjects and healthy controls at baseline assessment.

Category	Control	AD	*p*-Value
**Number of Subjects**	209	172	
**Gender (Male/Female)**	108/101	91/81	≥0.81 Chi Square
**Age**	75.87±5.10	75.31±7.32	≥0.39
**MMSE**	29.09±1.00	23.33±2.06	<0.0001
**ADASCog**	9.33±4.14	27.63±9.58	<0.0001
**Years of Education**	15.99±2.90	14.74±3.15	<0.0001
**Handedness (right/left)**	191/18	162/10	≥0.29 Chi Square
**Ethnicity (Hispanic/non-Hispanic/unknown)**	1/205/3	4/166/2	≥0.327 Fisher's exact
**Race (African American/Asian/More than one race/White)**	14/3/0/192	7/1/2/162	≥0.256 Fisher's exact
**APOE (ε2/ε3/ε4)**	34/322/62	8/194/142	<0.0001 Chi Square

### Case Control analysis

The APOE ε4 alleles were overrepresented in the AD cases as expected (Cochran-Armitage test χ^2^ (1 d.f.) = 62.3, p-value = 2.98×10^−15^). Of the two SNPs comprising the APOE genotype, rs429358 SNP was significant at p-value = 2.30×10^−16^ and rs7412 at p-value = 5.00×10^−4^ (see Table 2). The second most significant finding in the APOE region is the SNP rs2075650 (p-value = 7.47×10^−7^) located in intron 2 of TOMM40 gene. The haplotype association via *in silico* reconstruction of proximal SNPs increases the importance of the findings at this locus. One of the haplotypes constructed with rs2075650 and the two flanking SNPs rs11556505 (TOMM40) and rs429358 (APOE) has greater significance (p-value = 1.50×10^−11^) than the univariate p-value for the TOMM40 SNP.

**Table 2:** Case – control association results in the region of interest harboring APOE gene.

Gene	SNP	Coordinate (Build 36.3)	Type	Consequence Type	Allele frequency [%] cases/controls	*p*-Value
TOMM40	rs8102977	50085313	imputed	5′UTR	2.3/1.3	0.3
TOMM40	rs157580	50087106	genotyped	intronic	73.8/62.7	1.10×10^−3^
TOMM40	rs2075650	50087459	genotyped	intronic	29.9/15.1	7.48×10^−7^
TOMM40	rs11556505	50087984	imputed	synonymous-coding	26.5/14.3	2.96×10^−5^
TOMM40	rs8106922	50093506	genotyped	intronic	70.6/64.8	0.08
TOMM40	rs1160985	50095252	imputed	intronic	58.7/52.0	0.07
APOE	rs405509	50100676	genotyped	5′UTR	57.6/49.3	0.02
APOE	rs769451	50102751	genotyped	intronic	98.8/97.6	0.21
APOE	rs429358	50103781	genotyped	non-synonymous-coding	41.3/14.9	2.30×10^−16^
APOE	rs7412	50103919	genotyped	non-synonymous-coding	97.7/91.9	5.00×10^−4^
LOC100129500	rs439401	50106291	genotyped	intronic	75.0/62.9	4.00×10^−4^
LOC100129500-APOC1	rs5114	50110286	imputed	intronic	1.00/99.5	0.19
LOC100129500-APOC1	rs389261	50112183	imputed	intronic	98.3/96.4	0.15
LOC100129500-APOC1	rs10424339	50112333	imputed	intronic	99.0/98.9	0.92
LOC100129500-APOC1	rs12721054	50114427	imputed	intronic	1.00/99.5	0.2

Case control association analysis in the APOE region with special focus on TOMM40, APOE and APOC1 genes. Gene name, SNP name (rs number), SNP base-pair position (build 36.3), genotype type (either obtained through actual genotyping or through in *silico* imputation), physical location or SNP function (consequence type), case-control allele frequencies and p-values ordered by base-pair position.

### QT analysis

**Figure 1:**
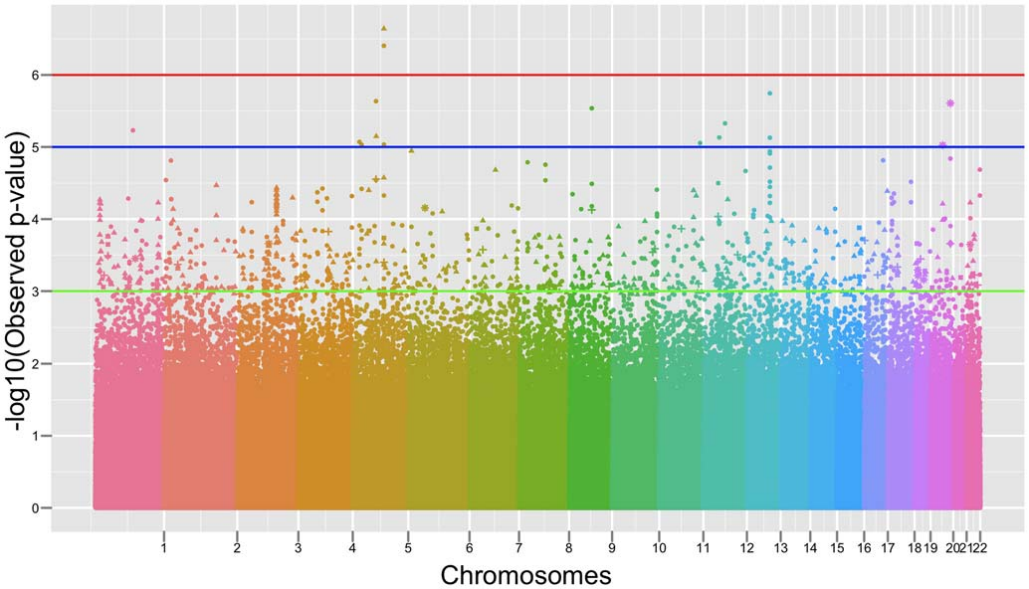
Manhattan plot of the quantitative trait genome wide association analysis. Manhattan plot of the p-values (-Log_10_(Observed p-value)) from the QT (right hippocampus) genome-wide association analysis. The spacing between the SNPs does not reflect the actual distances between SNPs in the genome. Each color identifies an autosomal chromosome (from chromosome 1 to chromosome 22). The horizontal lines display the cutoffs for 3 significance levels: green line for mild significance (10^−3^<p<10^−5^), blue line for high significance (10^−5^<p<10^−6^) and red line for genome-wide significance level (p<10^−6^). Different symbols reflect the function of the SNPs as detailed here: “▴” intronic, “▪” 3′UTR or downstream region, “+” 5′UTR or upstream region, “⊠” synonymous coding, “░” non-synonymous coding, “•” intergenic.

**Figure 2:**
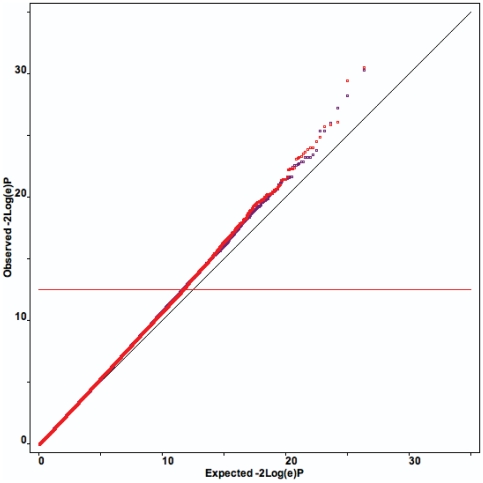
Q-Q plot of the quantitative trait genome wide association analysis. Q-Q plot of the quantitative trait genome wide association analysis for both right hippocampus (red dots) and left hippocampus (violet dots).

The QT analysis identified 25 SNPs significant at the level of≤10^−6^ associated with either the left or right hippocampal grey matter volume (see Figure 1). A Q-Q plot of the association results is presented in Figure 2. P-values from the observed distribution of the test of interest (i.e., the SNPxDiagnosis interaction) are plotted for all SNPs against the null distribution: p-values show a deviation in excess in the upper tail distribution, suggesting the presence of significant genetic association. Such deviation becomes evident at a p<1×10^−6^ supporting the hypothesis of a threshold for a claim of genetic association at this significance value. The significant SNPs (see Table 3) represent 15 loci and 6 desert regions. The QT analysis for all SNPs is provided in the supplementary materials (see Supplementary files). Of the 25 significant SNPs, thirteen map directly in genes and pseudogenes or are linked based on the LD structure of the region. Four SNPs map in regions characterized by a high level of genomic complexity given the overlap of several different genes and isoforms or pseudogenes in these areas. Eight SNPs map to 6 different “desert” regions where no specific gene annotation could be found including the LD block structure of the region. Details of these 25 significant SNPs follow. Supporting figures ( Figures S1 – Figure S20 ) and tables ( Table S1 – Table S20) are available for each of the 25 significant SNPs.

**Table 3:** Quantitative trait genome wide association top significant results.

Chromosome	Gene Context	SNP	Coordinate(Build 36.3)	Location	Genomic context - Distance to gene	p-value	Supporting Information
1	AL157404.18 - S100A5	rs4845552	151746622	INTERGENIC	7 Kbp from AL157404.18	6.23×10^−6^	Figure S1 - Table S1
5	AC026790.5	rs682748	17201911	INTERGENIC	9 Kbp from the upstram of AC026790.5	8.14×10^−6^	Figure S2 - Table S2
5	AC022418.5	rs7727656	25796436	INTERGENIC	desert region	8.27×10^−6^	Figure S3 - Table S3
5	AC104108.3 -SCAMP1 - LHFPL2	rs6881634	77666610	INTERGENIC	14 Kbp from AC104108.3	1.93×10^−6^	- Table S4
5	ARSB	rs337847	78295644	INTRONIC	intron 3	6.71×10^−6^	Figure S5 - Table S5
5	EFNA5	rs10074258	107010459	INTRONIC	intron1	2.15×10^−7^	Figure S6 - Table S6
5	EFNA5	rs12654281	107068341	INTERGENIC	30 Kbp from 5′UTR of EFNA5	3.72×10^−7^	Figure S6 - Table S6
5	EFNA 5	rs12657273	107074597	INTERGENIC	36 Kbp from 5′UTR of EFNA5	8.92×10^−6^	Figure S6 - Table S6
7	AC096553.4	rs9918508	9505804	INTERGENIC	desert region	8.49×10^−6^	Figure S7 - Table S7
7	IKZF1 - AC020743.7	rs2124799	50240560	INTERGENIC	desert region - 75 Kbp from IKFZ1	8.54×10^−6^	Figure S8 - Table S8
7	IKZF1 - AC020743.7	rs10276619	50283898	INTERGENIC	desert region - 31 Kbp from IKFZ1	2.94×10^−6^	Figure S8 - Table S8
7	MAGI2	rs11525066	78013913	INTRONIC	intron 3	2.85×10^−6^	Figure S9 - Table S9
8	MAL2	rs1364705	120293987	INTRONIC	intron 2	8.92×10^−6^	Figure S10 - Table S10
9	PRUNE2	rs10781380	78597964	INTRONIC	intron 6	7.13×10^−7^	Figure S11 - Table S11
9	RP11-232A1.1	rs10867752	83204857	INTERGENIC	8 Kbp from the upstream of RP11-232A1.1	3.08×10^−6^	Figure S12 - Table S12
11	ETS1	rs6590322	127711620	INTERGENIC	desert region	9.37×10^−6^	Figure S13 - Table S13
12	ARID2 - SFRS2IP	rs1373549	44772105	INTERGENIC	100 Kbp downstream SFRS2IP	7.80×10^−6^	Figure S14 - Table S14
12	CAND1	rs1082714	65915098	INTERGENIC	34 Kbp from 5′UTR of CAND1	4.93×10^−6^	Figure S15 - Table S15
13	RP11-506F17.1	rs9301535	85984283	INTERGENIC	desert region	7.90×10^−6^	Figure S16 - Table S16
13	RP11-506F17.1	rs4773460	86040858	INTERGENIC	desert region	1.93×10^−6^	Figure S16 - Table S16
14	FRMD6 - AL079307.7	rs11626056	51303026	UPSTREAM	upstream region of AL079307.7	1.18×10^−6^	Figure S17 - Table S17
20	C20orf132	rs8115854	35199751	NON_SYNONYMOUS_CODING	exon 11 - non-synonymous coding	2.09×10^−6^	Figure S18 - Table S18
20	RPN2	rs6031882	35243197	INTRONIC	intron 1	6.20×10^−6^	Figure S18 - Table S18
20	ZBP1	rs2073145	55624040	NON_SYNONYMOUS_CODING;SPLICE_SITE	exon 2 - non-synonymous coding; splice site	2.13×10^−6^	Figure S19 - Table S19
21	FDPSP	rs1888414	20699601	INTERGENIC	desert region - 16 Kbp from FDPSP	2.41×10^−7^	Figure S20 - Table S20

Quantitative trait analysis for the interaction between hippocampal grey matter volume (either left or right hemisphere) and SNPs from the genome-wide association study. All listed genes have at least one SNP in the left or right hippocampi at≤10^−6^. The chromosome number, gene context, SNP name (rs number), SNP base-pair position (build 36.3), physical location or SNP function (consequence type) are presented ordered by p-value.

Two of the most significant SNPs, rs10074258 and rs12654281, are both related to the gene EFNA5 (p-value = 2.15×10^−7^and 3.72×10^−7^ respectively). The LD pattern of this region allows the identification of a 61-Kbp LD block that harbors the 5′ end of the EFNA5 gene. Eleven out of the 16 SNPs in this genomic area are nominally significant (10^−6^<p<.05) providing additional support for the involvement of EFNA5 in AD. Six more nominally significant SNPs (10^−4^<p<10^−3^) are located in a ∼31-Kbp LD block on the same chromosome, 64 Kbp upstream the gene and containing the pseudogene AC024587.5 characterized by 2 isoforms partially overlapping in the genome. The two isoforms may have different functions according to the biotype description: the first one is a scRNA pseudogene and the second is miscellaneous pseudogene.

SNP rs10781380 is in the gene PRUNE2, which spans a total of 117-Kbp and includes 44 SNPs. An additional six nominally significant (10^−4^<p<10^−3^) SNPs are located in the same 41-Kbp LD block that includes 15 SNPs in introns 6, exon 7 and the downstream region of the gene. PRUNE2 has two partially overlapping isoforms.

SNP rs8115854 is associated at the GWAS threshold significance level of 10^−6^. This SNP is a non-synonymous coding polymorphism (I509T or I474T) mapping C20orf132 that shows 4 different isoforms and encodes for the hypothetical protein LOC140699. The C20orf132 gene spans a total of ∼78-Kbp and 7 SNPs were genotyped successfully in our sample. The most significant SNP (p-value = 2.09×10^−6^) is responsible for a threonine to isoleucine substitution. Three additional SNPs, rs8120307, rs1780682 and rs1744760 are nominally significant (10^−5^<p<10^−3^) and are located in intron 1 and 8 and exon 19 respectively. SNP rs1744760 (Q836R) (p-value = 1.7×10^−3^) is another non-synonymous (arginine-glutamine) coding SNP. This gene is located in a ∼230-kbp genomic region characterized by a high degree of LD that contains several overlapping different genes: RBL1 (2 isoforms), RPS3AP3, RPS27AP3, RP3-343K2.4 and RPN2 (7 isoforms). This genomic region includes 10 SNPs beside the 7 located in C20orf132. SNP rs6031882 (p-value = 6.20×10^−6^) is located in intron 1 of RPN2. Two out of these total 10 SNPs are also nominally significant (10^−5^<p<10^−3^): rs1892205 and rs3897903 are located in intron 2 and 12 of RBL1.

SNP rs2073145 (p-value = 2.13×10^−6^) is a non-synonymous coding polymorphism (E88K) responsible for a glutamic acid (E) by lysine (K) substitution in the ZBP1 gene that encodes a Z-DNA binding protein. This SNP is located in a 26-Kbp LD block that encompasses only the 5′ upstream region of the gene and where 7 out of 10 SNPs are nominally significant (10^−4^<p<.05).

SNP rs10867752 (p-value = 3.08×10^−6^) is located ∼8 kbp distal from the upstream of the pseudogene RP11-232A1.1 on chromosome 9. Its 27-Kbp LD block also contains 5 out of 8 SNPs nominally significant (10^−5^<p<10^−3^). The genomic region downstream from the pseudogene is characterized by a high degree of LD. The QC filters did not allow the calculation of the statistics for most of the SNPs that are included in this ∼73-Kbp LD block.

SNP rs1082714 (p-value = 4.93×10^−6^) is located 34-Kbp from the 5′ end of CAND1 gene on a large LD block that also includes the gene itself. Thus, a synonymous-coding SNP rs1060350 (L1183L) of CAND1 (p-value = 0.02) might acquire relevance.

Four other SNPs map to a similar number of genes, in all cases also showing other nominally significant SNPs in addition to those that present a genome-wide significance. In particular, SNP rs337847 is related to the ARSB gene, SNP rs682748 to the processed pseudogene AC026790.5 (with 9 out of 14 SNPs significant). SNP rs1364705 is related to the gene MAL2 and SNP rs11525066 to the gene MAGI2.

The four complex regions are characterized by the overlap of many different genes, isoforms of the same genes or pseudogenes. SNP rs6881634 (p-value = 1.93×10^−6^) identifies a locus of interest in an apparently conserved region of ∼190-Kbp in chromosome 5 containing several different pseudogenes and genes: AC104108.3, SCAMP1 (3 isoforms), LHFPL2 (2 isoforms). The most significant SNP is located ∼14-Kbp from AC104108.3 but is also in strong LD (D' = 1) with the first typed SNP located at the upstream of the SCAMP1 gene. This genomic region includes 13 other SNPs out of 33 that are nominally significant (10^−5^<p<10^−3^) beside the top SNP.

SNP rs11626056 (p-value = 1.18×10^−6^) is located in a 35-Kbp genomic region of chromosome 14 characterized by a very low degree of LD that harbors two isoforms of the same gene AL079307.7: a retrotransposed gene and a pseudogene. Due to the low degree of LD and the few SNPs typed that passed QC, it is not possible to link this SNP to any annotated genes. The genomic area surrounding this locus seems conserved based on the LD pattern, though, and might be of interest as it contains 2 additional isoforms of the same olfactory receptor pseudogene (OR7E) with different functions (i.e. rRNA and snRNA) overlapping with the gene FRMD6.

SNP rs4845552 (p-value = 6.23×10^−6^) on chromosome 1 is located ∼7-Kbp from the locus AL157404.18, hypothetically coding a transcription regulatory misc RNA. This misc RNA is located in a conserved region characterized by high LD that contains several additional genes and their different isoforms: S100A5 (3), S100A4 (4), S100A3 (2) and S100A2 (5). None of the SNPs belonging to the S100A family genes passed QC filters.

SNP rs1373549 (p-value = 7.80×10^−6^) on chromosome 12 is intergenic and identifies a locus of interest encompassing several different pseudogenes and genes: ARID2 (2 isoforms), SFRS2IP (4 isoforms) and the pseudogene AC009464.8. The most significant SNP is located ∼100-Kbp downstream SFRS2IP in a genomic region characterized by a high degree of LD. In the same region (from 44802396 bp to 44420742 bp) 22 out of 32 SNPs are nominally significant (10^−5^<p<10^−3^) beside the top SNP.

The 8 remaining top SNPs identify 6 loci of interest located in genomic regions characterized by the absence of known annotated genes, so called “desert” regions. Five out of the 8 significant findings are between 60 to to 200 Kbp from the closest annotated genes or pseudogenes. SNPs rs1888414 is located ∼16-Kbp and rs10276619 and rs2124799 are located ∼31-Kbp from the closest genes, FDPSP and IKFZ1 respectively. These SNPs, however, are not in an LD region for either gene.

## Discussion

The goal of this paper was to present an initial QT analysis combining imaging and genetic data obtained in a GWAS from the ADNI cohort, in addition to a standard case-control analysis. Our aim was to make publicly available genotype data for AD and controls in the context of rich multimodal imaging data and comprehensive clinical data that can be investigated by the research community, as well as conducting a preliminary analysis to identify potential genes related to the susceptibility of AD. The case-control analysis confirmed the association with APOE alleles, as expected, and also implicated TOMM40, a gene physically close to APOE that contributes additional risk to developing AD [31], [32] (TOMM40, translocase of outer mitochondrial membrane 40) influences mitochondrial function. Aging both decreases the number of mitochondria and increases risk of developing AD. TOMM40 alleles have been recently linked to an earlier onset of AD (Roses AD et al.: “Apoe-3 And Tomm-40 Haplotypes Determine Inheritance Of Alzheimer's Disease Independently Of Apoe-4 Risk”. Alzheimer's Association 2009 International Conference on Alzheimer's Disease, July 12, 2009).

The application of a QT analysis for the interaction of genotypes with diagnosis, however, allowed us to identify novel candidate genes at high levels of genome-wide significant SNPs. We found a strong main effect for the APOE ε4 allele on the hippocampal volume. However, we did not find a diagnosis by allele interaction relative to APOE ε4, suggesting that APOE alleles affect hippocampal volume similarly in both AD patients and healthy controls. This is consistent with most, but not all, of the literature finding an association of APOE ε4 with accelerated age-related volume loss in hippocampus and several other brain regions as well as in AD [33]–[36]. These results suggest an effect of APOE 4 on the brain atrophy, independent from its overrepresentation in AD.

The rationale of this study is that hippocampal atrophy in AD subjects is more objective and closer to the genes contributing to AD than the clinical subjective diagnosis of AD alone. Hippocampal atrophy is characterized by neuronal loss and decreased synaptic density and is believed to be caused by the accumulation of neuropathological events in addition to the observed amyloid and tau pathology [37]. Apoptosis, cell cycle impairment and the alteration of protein folding and degradation through ubiquination are among the candidate pathophysiological mechanisms that may underlie neurodegeneration in AD [38]–[45]. Based on our preliminary functional annotation, our significant SNPs related to genes PRUNE2, MAGI2, ARSB, EFNA5, CAND1 may be associated with these biochemical and cellular changes.

We found associations between hippocampal volume reductions in AD subjects and the EFNA5 (ephrin-A5), ARSB (arylsulfatase B), MAGI2 (membrane associated guanylate kinase, WW and PDZ domain containing 2), PRUNE2 (prune homolog 2), and CAND1 (cullin-associated and neddylation-dissociated 1) genes. These genes are associated with the aforementioned biochemical processes, namely ubiquination (MAGI2, CAND1), apoptosis (CAND1, PRUNE2), oxidative necrosis (ARSB), hippocampal development (EFNA5), and dementia, (MAGI2, ARBS). The presence of the highly conserved BCH domain (BNIP2 and Cdc42GAP homology) in PRUNE2 which is involved in a variety of protein-protein interactions and the presence of the BNIP-2 domain, a substrate for caspase cleavage suggest a role for PRUNE2 in apoptosis [46]. CAND1 allows the complex formation between CUL1 and SKP [47]. Cullins (CUL) are scaffold proteins for the ubiquitin ligase complex, and neddylation of cullin enhances its ability to promote ubiquitination [48]. NEDD8 has been found in ubiquitinated neurofibrillary tangles in the AD brain [49]. The conjugation of NEDD8 is stimulated by the interaction of APP with APP-BP1 that mediates APP-induced apoptosis in the AD brain [50].

MAGI2, together with ATN1 (atrophin 1), has been implicated in Dentato-Rubral and PallidoLuysian Atrophy (DRPLA), a disease with a syndrome of myoclonic epilepsy, dementia, ataxia, and choreoathetosis [51]. MAGI2 has been speculated to play a critical role in the ubiquitination process of ATN1 by linking ubiquitinating enzyme to plasma membrane proteins, neurotransmitter receptors and ion channels [51]. Perturbation of the ubiquitin system could potentially lead to increased misfolded protein accumulation and cell cycle abnormalities, and apoptosis [52].

The gene EFNA5 is highly expressed in the human brain and hippocampus. Ephrin-A5 is a member of the Ephrin (Eph) superfamily implicated in mediating developmental events in the nervous system [53], [54]. The Ephrin family of receptors regulates cell morphology and behavior by rearranging the actin cytoskeleton through the Rho family of small G-proteins [55]. Eph-A receptor signaling has been directly implicated in axon guidance, while Eph-B receptor signaling has been linked to dendritic spine morphogenesis [56]. EFNA5 and its interactions with other axon guidance genes have been linked to susceptibility to developing Parkinson's disease [57]. It is possible that the EFNA5 findings are consistent with a neurodevelopmental process in both AD and Parkinson's disease.

ARSB (Arylsulfatase B) is a lysosomal enzyme that removes the sulfate ester group of *N*-acetylgalactosamine-4-sulfate in dermatan and chondroitin sulfate. A mutation in this enzyme leads to the development of the lysosome storage disease called mucopolysaccharidosis VI [58]. The accumulation of amyloid *β* peptide (A*\*) is toxic to neuronal cells by inducing oxidative stress [59]. In response to oxidative stress, alterations of various endosomal-lysosomal components including ARSB have been observed in AD brains [60], [61]. This fact could support the hypothesis that ARSB is involved in the modulation of the oxidative stress pathway and neuronal loss in the development of AD.

Our results highlight the variety of data that can be obtained from these different analytic approaches. Previous GWASs in AD using a categorical approach have not been able to consistently identify risk genes in AD other than APOE [62]. To further evaluate our finding of no interaction with diagnosis and APOE we performed a QT analysis removing APOE–related SNPs. The results of the analysis were very similar with the addition of a single SNP, rs8027305 in THSD4, significant at p≤10^−6^.

It is important to emphasize that the SNPs from the Illumina Human610-Quad BeadChip are tagging SNPs and as such represents “flags” for a possible location of a susceptibility locus to AD, and rarely – if ever – are the causal SNPs. The advantage of a tagging SNPs approach is that it maps an average 10 to max 20 kilobases in which a causal DNA variant may be found, thus indicating an area of focus for gene sequencing and subsequent studies of molecular mechanisms. The genes that we identified by surrogate SNPs are candidates that require additional confirmation in independent samples. Our method using brain imaging as a quantitative trait is fundamentally different from case-control approaches as it begins with brain imaging as a dependent variable and then identifies as genes that affect the brain imaging differences between AD and healthy controls. This is a very different contrast than comparing frequencies of genotypes across cases and controls and therefore is not expected to identify the same results as a QT approach. Interestingly, some of our findings are located in putative hot spots regions for AD, previously identified by other investigators using case-control, linkage and other methods. These overlapping hot spots include regions on chromosomes 5 [63], 9 [64]–[66], and 14 [67].

The sample only contained mild AD patients, a relatively narrow range of illness, and therefore is not fully representative of the disease. Also, the ADNI sample was not collected under an epidemiological ascertainment strategy and the sample size itself is small, further limiting its generalizability. The small sample size, however, was somewhat mitigated by the four to eightfold increase in statistical power of a quantitative trait design over a categorical case-control analysis [4], [68]. Our case control analysis suffered from inadequate power relative to the more powered QT analysis. Nevertheless, the possibility of false positives remains. Also, there is no a definitive established method for determining appropriate statistical threshold values for an interaction term (i.e., SNP×diagnosis) in a QT analysis in the context of a genome-wide scan. Our solution was to consider the physical clustering of SNPs and their biological plausibility and to report the full extent of our findings in the paper and supplemental tables. This allows the reader to consider our preliminary results in terms of their statistical and biological consistency given the multiple testing issues. This is similar to the strategy used by Almasy et al. 2008 [69].

Hippocampal volume was chosen as our quantitative phenotype because of its sensitivity to the changes of early AD disease [70]. Clinical diagnosis of AD in part depends on subjective reporting by the patient and caretaker. The clinical presentation may be more distal from the underlying genetic causes than the brain atrophy that usually predates symptoms by many years. Identifying genes that contribute to hippocampal atrophy have the potential to identify novel genes and pathways involved in the development of Alzheimer's disease at its earliest stages. It is possible that other brain imaging phenotypes would identify other candidate genes.

### Conclusion

This QT study is a cross-sectional analysis of baseline hippocampal grey matter density with common genetic variants as measured by SNP genotypes in the ADNI sample. A QT analysis provides a powerful strategy for gene discovery in the context of a genome-wide survey. ADNI provides longitudinal clinical, cognitive, biomarker and imaging data which will facilitate the search for genes that are associated with conversion from normal to MCI and from MCI to AD, as well as the rates of change within and between these categories. The goal of this preliminary analysis was to identify unanticipated risk genes for the development of mild AD as represented by hippocampal atrophy. Elucidating the roles of PRUNE2, MAGI2, ARSB and EFNA5 in the regulation of neurodevelopment, protein degradation, apoptosis and neuronal loss could enhance our understanding of hippocampal atrophy and AD. This method of gene discovery complements other established strategies such as case-control designs with large sample sizes, family-based design, and targeted candidate gene approaches. To address the concerns of multiple testing and false positive findings, all genotyped data are publicly available (LONI, http://www.loni.ucla.edu/ADNI/Data/) for combining with other datasets and for other analytical approaches by the scientific community.

## Supporting Information

Figure S1 QT analysis of SNPs associated with genes or chromosomal regions as reported in Table 3 of the manuscript. Physical map of the SNPs associated with genes or chromosomal regions in the ADNI sample produced by WGAViewer. The top of the figure is the ideogram of the chromosome; the vertical red line depicts the relative location of locus of interest. Below the graph are the -log p significance values of the individual SNPs on the imaging phenotype (hippocampal atrophy) for the left and right hemispheres as indicated in each figure. The blue lines below the graph indicate the location of the exons in the transcripts annotated (translated region of the DNA). The vertical lines above the accompanying triangular matrix indicate the SNP locations, and demonstrate the LD pattern between SNPs (D'). The warmer colors on the flame scale indicate greater LD while the blue indicates absence of LD.
(0.80 MB TIF)

Figure S2 QT analysis of SNPs associated with genes or chromosomal regions as reported in Table 3 of the manuscript. Physical map of the SNPs associated with genes or chromosomal regions in the ADNI sample produced by WGAViewer. The top of the figure is the ideogram of the chromosome; the vertical red line depicts the relative location of locus of interest. Below the graph are the -log p significance values of the individual SNPs on the imaging phenotype (hippocampal atrophy) for the left and right hemispheres as indicated in each figure. The blue lines below the graph indicate the location of the exons in the transcripts annotated (translated region of the DNA). The vertical lines above the accompanying triangular matrix indicate the SNP locations, and demonstrate the LD pattern between SNPs (D'). The warmer colors on the flame scale indicate greater LD while the blue indicates absence of LD.
(0.85 MB TIF)

Figure S3 QT analysis of SNPs associated with genes or chromosomal regions as reported in Table 3 of the manuscript. Physical map of the SNPs associated with genes or chromosomal regions in the ADNI sample produced by WGAViewer. The top of the figure is the ideogram of the chromosome; the vertical red line depicts the relative location of locus of interest. Below the graph are the -log p significance values of the individual SNPs on the imaging phenotype (hippocampal atrophy) for the left and right hemispheres as indicated in each figure. The blue lines below the graph indicate the location of the exons in the transcripts annotated (translated region of the DNA). The vertical lines above the accompanying triangular matrix indicate the SNP locations, and demonstrate the LD pattern between SNPs (D'). The warmer colors on the flame scale indicate greater LD while the blue indicates absence of LD.
(0.71 MB TIF)

Figure S4 QT analysis of SNPs associated with genes or chromosomal regions as reported in Table 3 of the manuscript. Physical map of the SNPs associated with genes or chromosomal regions in the ADNI sample produced by WGAViewer. The top of the figure is the ideogram of the chromosome; the vertical red line depicts the relative location of locus of interest. Below the graph are the -log p significance values of the individual SNPs on the imaging phenotype (hippocampal atrophy) for the left and right hemispheres as indicated in each figure. The blue lines below the graph indicate the location of the exons in the transcripts annotated (translated region of the DNA). The vertical lines above the accompanying triangular matrix indicate the SNP locations, and demonstrate the LD pattern between SNPs (D'). The warmer colors on the flame scale indicate greater LD while the blue indicates absence of LD.
(0.87 MB TIF)

Figure S5 QT analysis of SNPs associated with genes or chromosomal regions as reported in Table 3 of the manuscript. Physical map of the SNPs associated with genes or chromosomal regions in the ADNI sample produced by WGAViewer. The top of the figure is the ideogram of the chromosome; the vertical red line depicts the relative location of locus of interest. Below the graph are the -log p significance values of the individual SNPs on the imaging phenotype (hippocampal atrophy) for the left and right hemispheres as indicated in each figure. The blue lines below the graph indicate the location of the exons in the transcripts annotated (translated region of the DNA). The vertical lines above the accompanying triangular matrix indicate the SNP locations, and demonstrate the LD pattern between SNPs (D'). The warmer colors on the flame scale indicate greater LD while the blue indicates absence of LD.
(0.95 MB TIF)

Figure S6 QT analysis of SNPs associated with genes or chromosomal regions as reported in Table 3 of the manuscript. Physical map of the SNPs associated with genes or chromosomal regions in the ADNI sample produced by WGAViewer. The top of the figure is the ideogram of the chromosome; the vertical red line depicts the relative location of locus of interest. Below the graph are the -log p significance values of the individual SNPs on the imaging phenotype (hippocampal atrophy) for the left and right hemispheres as indicated in each figure. The blue lines below the graph indicate the location of the exons in the transcripts annotated (translated region of the DNA). The vertical lines above the accompanying triangular matrix indicate the SNP locations, and demonstrate the LD pattern between SNPs (D'). The warmer colors on the flame scale indicate greater LD while the blue indicates absence of LD.
(0.85 MB TIF)

Figure S7 QT analysis of SNPs associated with genes or chromosomal regions as reported in Table 3 of the manuscript. Physical map of the SNPs associated with genes or chromosomal regions in the ADNI sample produced by WGAViewer. The top of the figure is the ideogram of the chromosome; the vertical red line depicts the relative location of locus of interest. Below the graph are the -log p significance values of the individual SNPs on the imaging phenotype (hippocampal atrophy) for the left and right hemispheres as indicated in each figure. The blue lines below the graph indicate the location of the exons in the transcripts annotated (translated region of the DNA). The vertical lines above the accompanying triangular matrix indicate the SNP locations, and demonstrate the LD pattern between SNPs (D'). The warmer colors on the flame scale indicate greater LD while the blue indicates absence of LD.
(0.87 MB TIF)

Figure S8 QT analysis of SNPs associated with genes or chromosomal regions as reported in Table 3 of the manuscript. Physical map of the SNPs associated with genes or chromosomal regions in the ADNI sample produced by WGAViewer. The top of the figure is the ideogram of the chromosome; the vertical red line depicts the relative location of locus of interest. Below the graph are the -log p significance values of the individual SNPs on the imaging phenotype (hippocampal atrophy) for the left and right hemispheres as indicated in each figure. The blue lines below the graph indicate the location of the exons in the transcripts annotated (translated region of the DNA). The vertical lines above the accompanying triangular matrix indicate the SNP locations, and demonstrate the LD pattern between SNPs (D'). The warmer colors on the flame scale indicate greater LD while the blue indicates absence of LD.
(0.90 MB TIF)

Figure S9 QT analysis of SNPs associated with genes or chromosomal regions as reported in Table 3 of the manuscript. Physical map of the SNPs associated with genes or chromosomal regions in the ADNI sample produced by WGAViewer. The top of the figure is the ideogram of the chromosome; the vertical red line depicts the relative location of locus of interest. Below the graph are the -log p significance values of the individual SNPs on the imaging phenotype (hippocampal atrophy) for the left and right hemispheres as indicated in each figure. The blue lines below the graph indicate the location of the exons in the transcripts annotated (translated region of the DNA). The vertical lines above the accompanying triangular matrix indicate the SNP locations, and demonstrate the LD pattern between SNPs (D'). The warmer colors on the flame scale indicate greater LD while the blue indicates absence of LD.
(0.82 MB TIF)

Figure S10 QT analysis of SNPs associated with genes or chromosomal regions as reported in Table 3 of the manuscript. Physical map of the SNPs associated with genes or chromosomal regions in the ADNI sample produced by WGAViewer. The top of the figure is the ideogram of the chromosome; the vertical red line depicts the relative location of locus of interest. Below the graph are the -log p significance values of the individual SNPs on the imaging phenotype (hippocampal atrophy) for the left and right hemispheres as indicated in each figure. The blue lines below the graph indicate the location of the exons in the transcripts annotated (translated region of the DNA). The vertical lines above the accompanying triangular matrix indicate the SNP locations, and demonstrate the LD pattern between SNPs (D'). The warmer colors on the flame scale indicate greater LD while the blue indicates absence of LD.
(0.57 MB TIF)

Figure S11 QT analysis of SNPs associated with genes or chromosomal regions as reported in Table 3 of the manuscript. Physical map of the SNPs associated with genes or chromosomal regions in the ADNI sample produced by WGAViewer. The top of the figure is the ideogram of the chromosome; the vertical red line depicts the relative location of locus of interest. Below the graph are the -log p significance values of the individual SNPs on the imaging phenotype (hippocampal atrophy) for the left and right hemispheres as indicated in each figure. The blue lines below the graph indicate the location of the exons in the transcripts annotated (translated region of the DNA). The vertical lines above the accompanying triangular matrix indicate the SNP locations, and demonstrate the LD pattern between SNPs (D'). The warmer colors on the flame scale indicate greater LD while the blue indicates absence of LD.
(1.01 MB TIF)

Figure S12 QT analysis of SNPs associated with genes or chromosomal regions as reported in Table 3 of the manuscript. Physical map of the SNPs associated with genes or chromosomal regions in the ADNI sample produced by WGAViewer. The top of the figure is the ideogram of the chromosome; the vertical red line depicts the relative location of locus of interest. Below the graph are the -log p significance values of the individual SNPs on the imaging phenotype (hippocampal atrophy) for the left and right hemispheres as indicated in each figure. The blue lines below the graph indicate the location of the exons in the transcripts annotated (translated region of the DNA). The vertical lines above the accompanying triangular matrix indicate the SNP locations, and demonstrate the LD pattern between SNPs (D'). The warmer colors on the flame scale indicate greater LD while the blue indicates absence of LD.
(0.78 MB TIF)

Figure S13 QT analysis of SNPs associated with genes or chromosomal regions as reported in Table 3 of the manuscript. Physical map of the SNPs associated with genes or chromosomal regions in the ADNI sample produced by WGAViewer. The top of the figure is the ideogram of the chromosome; the vertical red line depicts the relative location of locus of interest. Below the graph are the -log p significance values of the individual SNPs on the imaging phenotype (hippocampal atrophy) for the left and right hemispheres as indicated in each figure. The blue lines below the graph indicate the location of the exons in the transcripts annotated (translated region of the DNA). The vertical lines above the accompanying triangular matrix indicate the SNP locations, and demonstrate the LD pattern between SNPs (D'). The warmer colors on the flame scale indicate greater LD while the blue indicates absence of LD.
(0.95 MB TIF)

Figure S14 QT analysis of SNPs associated with genes or chromosomal regions as reported in Table 3 of the manuscript. Physical map of the SNPs associated with genes or chromosomal regions in the ADNI sample produced by WGAViewer. The top of the figure is the ideogram of the chromosome; the vertical red line depicts the relative location of locus of interest. Below the graph are the -log p significance values of the individual SNPs on the imaging phenotype (hippocampal atrophy) for the left and right hemispheres as indicated in each figure. The blue lines below the graph indicate the location of the exons in the transcripts annotated (translated region of the DNA). The vertical lines above the accompanying triangular matrix indicate the SNP locations, and demonstrate the LD pattern between SNPs (D'). The warmer colors on the flame scale indicate greater LD while the blue indicates absence of LD.
(1.20 MB TIF)

Figure S15 QT analysis of SNPs associated with genes or chromosomal regions as reported in Table 3 of the manuscript. Physical map of the SNPs associated with genes or chromosomal regions in the ADNI sample produced by WGAViewer. The top of the figure is the ideogram of the chromosome; the vertical red line depicts the relative location of locus of interest. Below the graph are the -log p significance values of the individual SNPs on the imaging phenotype (hippocampal atrophy) for the left and right hemispheres as indicated in each figure. The blue lines below the graph indicate the location of the exons in the transcripts annotated (translated region of the DNA). The vertical lines above the accompanying triangular matrix indicate the SNP locations, and demonstrate the LD pattern between SNPs (D'). The warmer colors on the flame scale indicate greater LD while the blue indicates absence of LD.
(0.82 MB TIF)

Figure S16 QT analysis of SNPs associated with genes or chromosomal regions as reported in Table 3 of the manuscript. Physical map of the SNPs associated with genes or chromosomal regions in the ADNI sample produced by WGAViewer. The top of the figure is the ideogram of the chromosome; the vertical red line depicts the relative location of locus of interest. Below the graph are the -log p significance values of the individual SNPs on the imaging phenotype (hippocampal atrophy) for the left and right hemispheres as indicated in each figure. The blue lines below the graph indicate the location of the exons in the transcripts annotated (translated region of the DNA). The vertical lines above the accompanying triangular matrix indicate the SNP locations, and demonstrate the LD pattern between SNPs (D'). The warmer colors on the flame scale indicate greater LD while the blue indicates absence of LD.
(0.93 MB TIF)

Figure S17 QT analysis of SNPs associated with genes or chromosomal regions as reported in Table 3 of the manuscript. Physical map of the SNPs associated with genes or chromosomal regions in the ADNI sample produced by WGAViewer. The top of the figure is the ideogram of the chromosome; the vertical red line depicts the relative location of locus of interest. Below the graph are the -log p significance values of the individual SNPs on the imaging phenotype (hippocampal atrophy) for the left and right hemispheres as indicated in each figure. The blue lines below the graph indicate the location of the exons in the transcripts annotated (translated region of the DNA). The vertical lines above the accompanying triangular matrix indicate the SNP locations, and demonstrate the LD pattern between SNPs (D'). The warmer colors on the flame scale indicate greater LD while the blue indicates absence of LD.
(0.79 MB TIF)

Figure S18 QT analysis of SNPs associated with genes or chromosomal regions as reported in Table 3 of the manuscript. Physical map of the SNPs associated with genes or chromosomal regions in the ADNI sample produced by WGAViewer. The top of the figure is the ideogram of the chromosome; the vertical red line depicts the relative location of locus of interest. Below the graph are the -log p significance values of the individual SNPs on the imaging phenotype (hippocampal atrophy) for the left and right hemispheres as indicated in each figure. The blue lines below the graph indicate the location of the exons in the transcripts annotated (translated region of the DNA). The vertical lines above the accompanying triangular matrix indicate the SNP locations, and demonstrate the LD pattern between SNPs (D'). The warmer colors on the flame scale indicate greater LD while the blue indicates absence of LD.
(0.98 MB TIF)

Figure S19 QT analysis of SNPs associated with genes or chromosomal regions as reported in Table 3 of the manuscript. Physical map of the SNPs associated with genes or chromosomal regions in the ADNI sample produced by WGAViewer. The top of the figure is the ideogram of the chromosome; the vertical red line depicts the relative location of locus of interest. Below the graph are the -log p significance values of the individual SNPs on the imaging phenotype (hippocampal atrophy) for the left and right hemispheres as indicated in each figure. The blue lines below the graph indicate the location of the exons in the transcripts annotated (translated region of the DNA). The vertical lines above the accompanying triangular matrix indicate the SNP locations, and demonstrate the LD pattern between SNPs (D'). The warmer colors on the flame scale indicate greater LD while the blue indicates absence of LD.
(0.90 MB TIF)

Figure S20 QT analysis of SNPs associated with genes or chromosomal regions as reported in Table 3 of the manuscript. Physical map of the SNPs associated with genes or chromosomal regions in the ADNI sample produced by WGAViewer. The top of the figure is the ideogram of the chromosome; the vertical red line depicts the relative location of locus of interest. Below the graph are the -log p significance values of the individual SNPs on the imaging phenotype (hippocampal atrophy) for the left and right hemispheres as indicated in each figure. The blue lines below the graph indicate the location of the exons in the transcripts annotated (translated region of the DNA). The vertical lines above the accompanying triangular matrix indicate the SNP locations, and demonstrate the LD pattern between SNPs (D'). The warmer colors on the flame scale indicate greater LD while the blue indicates absence of LD.
(0.90 MB TIF)

Table S1 Quantitative trait analysis for the interaction between left and right hippocampi for the SNPs and related genes or chromosomal regions listed in Table 3 of the manuscript. The SNP chromosome, gene, base-pair position, rs number, consequence type and SNP alleles are presented. The p-values for the left and right hippocampi indicate the interaction between SNP and diagnosis in explaining the subjects' degree of hippocampal atrophy.
(0.03 MB XLS)

Table S2 Quantitative trait analysis for the interaction between left and right hippocampi for the SNPs and related genes or chromosomal regions listed in Table 3 of the manuscript. The SNP chromosome, gene, base-pair position, rs number, consequence type and SNP alleles are presented. The p-values for the left and right hippocampi indicate the interaction between SNP and diagnosis in explaining the subjects' degree of hippocampal atrophy.
(0.04 MB XLS)

Table S3 Quantitative trait analysis for the interaction between left and right hippocampi for the SNPs and related genes or chromosomal regions listed in Table 3 of the manuscript. The SNP chromosome, gene, base-pair position, rs number, consequence type and SNP alleles are presented. The p-values for the left and right hippocampi indicate the interaction between SNP and diagnosis in explaining the subjects' degree of hippocampal atrophy.
(0.03 MB XLS)

Table S4 Quantitative trait analysis for the interaction between left and right hippocampi for the SNPs and related genes or chromosomal regions listed in Table 3 of the manuscript. The SNP chromosome, gene, base-pair position, rs number, consequence type and SNP alleles are presented. The p-values for the left and right hippocampi indicate the interaction between SNP and diagnosis in explaining the subjects' degree of hippocampal atrophy.
(0.04 MB XLS)

Table S5 Quantitative trait analysis for the interaction between left and right hippocampi for the SNPs and related genes or chromosomal regions listed in Table 3 of the manuscript. The SNP chromosome, gene, base-pair position, rs number, consequence type and SNP alleles are presented. The p-values for the left and right hippocampi indicate the interaction between SNP and diagnosis in explaining the subjects' degree of hippocampal atrophy.
(0.04 MB XLS)

Table S6 Quantitative trait analysis for the interaction between left and right hippocampi for the SNPs and related genes or chromosomal regions listed in Table 3 of the manuscript. The SNP chromosome, gene, base-pair position, rs number, consequence type and SNP alleles are presented. The p-values for the left and right hippocampi indicate the interaction between SNP and diagnosis in explaining the subjects' degree of hippocampal atrophy.
(0.04 MB XLS)

Table S7 Quantitative trait analysis for the interaction between left and right hippocampi for the SNPs and related genes or chromosomal regions listed in Table 3 of the manuscript. The SNP chromosome, gene, base-pair position, rs number, consequence type and SNP alleles are presented. The p-values for the left and right hippocampi indicate the interaction between SNP and diagnosis in explaining the subjects' degree of hippocampal atrophy.
(0.04 MB XLS)

Table S8 Quantitative trait analysis for the interaction between left and right hippocampi for the SNPs and related genes or chromosomal regions listed in Table 3 of the manuscript. The SNP chromosome, gene, base-pair position, rs number, consequence type and SNP alleles are presented. The p-values for the left and right hippocampi indicate the interaction between SNP and diagnosis in explaining the subjects' degree of hippocampal atrophy.
(0.04 MB XLS)

Table S9 Quantitative trait analysis for the interaction between left and right hippocampi for the SNPs and related genes or chromosomal regions listed in Table 3 of the manuscript. The SNP chromosome, gene, base-pair position, rs number, consequence type and SNP alleles are presented. The p-values for the left and right hippocampi indicate the interaction between SNP and diagnosis in explaining the subjects' degree of hippocampal atrophy.
(0.04 MB XLS)

Table S10 Quantitative trait analysis for the interaction between left and right hippocampi for the SNPs and related genes or chromosomal regions listed in Table 3 of the manuscript. The SNP chromosome, gene, base-pair position, rs number, consequence type and SNP alleles are presented. The p-values for the left and right hippocampi indicate the interaction between SNP and diagnosis in explaining the subjects' degree of hippocampal atrophy.
(0.03 MB XLS)

Table S11 Quantitative trait analysis for the interaction between left and right hippocampi for the SNPs and related genes or chromosomal regions listed in Table 3 of the manuscript. The SNP chromosome, gene, base-pair position, rs number, consequence type and SNP alleles are presented. The p-values for the left and right hippocampi indicate the interaction between SNP and diagnosis in explaining the subjects' degree of hippocampal atrophy.
(0.04 MB XLS)

Table S12 Quantitative trait analysis for the interaction between left and right hippocampi for the SNPs and related genes or chromosomal regions listed in Table 3 of the manuscript. The SNP chromosome, gene, base-pair position, rs number, consequence type and SNP alleles are presented. The p-values for the left and right hippocampi indicate the interaction between SNP and diagnosis in explaining the subjects' degree of hippocampal atrophy.
(0.04 MB XLS)

Table S13 Quantitative trait analysis for the interaction between left and right hippocampi for the SNPs and related genes or chromosomal regions listed in Table 3 of the manuscript. The SNP chromosome, gene, base-pair position, rs number, consequence type and SNP alleles are presented. The p-values for the left and right hippocampi indicate the interaction between SNP and diagnosis in explaining the subjects' degree of hippocampal atrophy.
(0.04 MB XLS)

Table S14 Quantitative trait analysis for the interaction between left and right hippocampi for the SNPs and related genes or chromosomal regions listed in Table 3 of the manuscript. The SNP chromosome, gene, base-pair position, rs number, consequence type and SNP alleles are presented. The p-values for the left and right hippocampi indicate the interaction between SNP and diagnosis in explaining the subjects' degree of hippocampal atrophy.
(0.04 MB XLS)

Table S15 Quantitative trait analysis for the interaction between left and right hippocampi for the SNPs and related genes or chromosomal regions listed in Table 3 of the manuscript. The SNP chromosome, gene, base-pair position, rs number, consequence type and SNP alleles are presented. The p-values for the left and right hippocampi indicate the interaction between SNP and diagnosis in explaining the subjects' degree of hippocampal atrophy.
(0.04 MB XLS)

Table S16 Quantitative trait analysis for the interaction between left and right hippocampi for the SNPs and related genes or chromosomal regions listed in Table 3 of the manuscript. The SNP chromosome, gene, base-pair position, rs number, consequence type and SNP alleles are presented. The p-values for the left and right hippocampi indicate the interaction between SNP and diagnosis in explaining the subjects' degree of hippocampal atrophy.
(0.04 MB XLS)

Table S17 Quantitative trait analysis for the interaction between left and right hippocampi for the SNPs and related genes or chromosomal regions listed in Table 3 of the manuscript. The SNP chromosome, gene, base-pair position, rs number, consequence type and SNP alleles are presented. The p-values for the left and right hippocampi indicate the interaction between SNP and diagnosis in explaining the subjects' degree of hippocampal atrophy.
(0.04 MB XLS)

Table S18 Quantitative trait analysis for the interaction between left and right hippocampi for the SNPs and related genes or chromosomal regions listed in Table 3 of the manuscript. The SNP chromosome, gene, base-pair position, rs number, consequence type and SNP alleles are presented. The p-values for the left and right hippocampi indicate the interaction between SNP and diagnosis in explaining the subjects' degree of hippocampal atrophy.
(0.04 MB XLS)

Table S19 Quantitative trait analysis for the interaction between left and right hippocampi for the SNPs and related genes or chromosomal regions listed in Table 3 of the manuscript. The SNP chromosome, gene, base-pair position, rs number, consequence type and SNP alleles are presented. The p-values for the left and right hippocampi indicate the interaction between SNP and diagnosis in explaining the subjects' degree of hippocampal atrophy.
(0.04 MB XLS)

Table S20 Quantitative trait analysis for the interaction between left and right hippocampi for the SNPs and related genes or chromosomal regions listed in Table 3 of the manuscript. The SNP chromosome, gene, base-pair position, rs number, consequence type and SNP alleles are presented. The p-values for the left and right hippocampi indicate the interaction between SNP and diagnosis in explaining the subjects' degree of hippocampal atrophy.
(0.04 MB XLS)
