# Supplementary figures and images for: Hippocampal Atrophy as a Quantitative Trait in a Genome-Wide Association Study Identifying Novel Susceptibility Genes for Alzheimer's Disease
Source: PLoS One. 2009 Aug 7;4(8):e6501. doi: 10.1371/journal.pone.0006501 (PMC2719581; doi:10.1371/journal.pone.0006501)

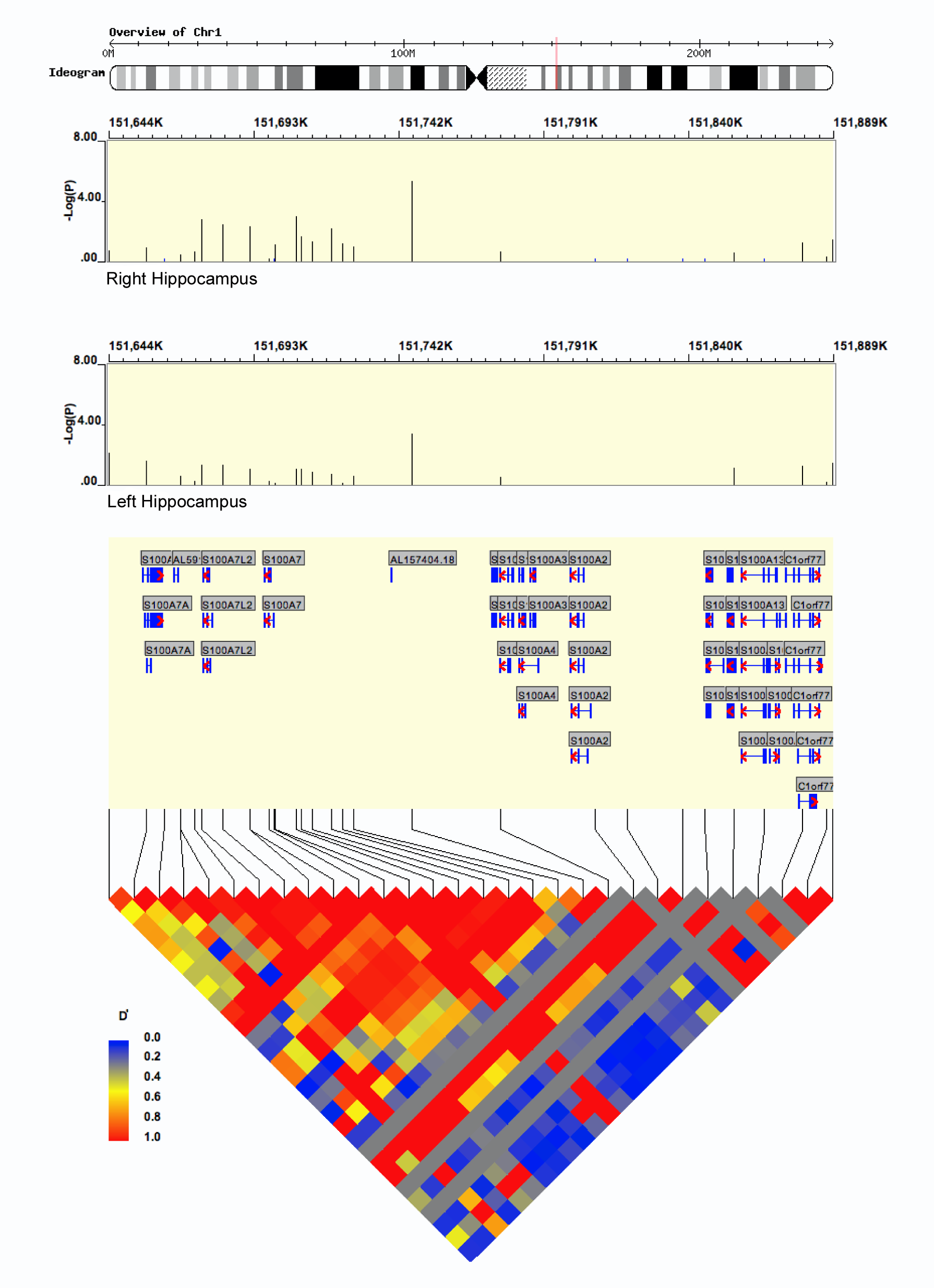

Supplement: Figure S1 — QT analysis of SNPs associated with genes or chromosomal regions as reported in Table 3 of the manuscript. Physical map of the SNPs associated with genes or chromosomal regions in the ADNI sample produced by WGAViewer. The top of the figure is the ideogram of the chromosome; the vertical red line depicts the relative location of locus of interest. Below the graph are the -log p significance values of the individual SNPs on the imaging phenotype (hippocampal atrophy) for the left and right hemispheres as indicated in each figure. The blue lines below the graph indicate the location of the exons in the transcripts annotated (translated region of the DNA). The vertical lines above the accompanying triangular matrix indicate the SNP locations, and demonstrate the LD pattern between SNPs (D'). The warmer colors on the flame scale indicate greater LD while the blue indicates absence of LD. (0.80 MB TIF) [file pone.0006501.s001.tif]

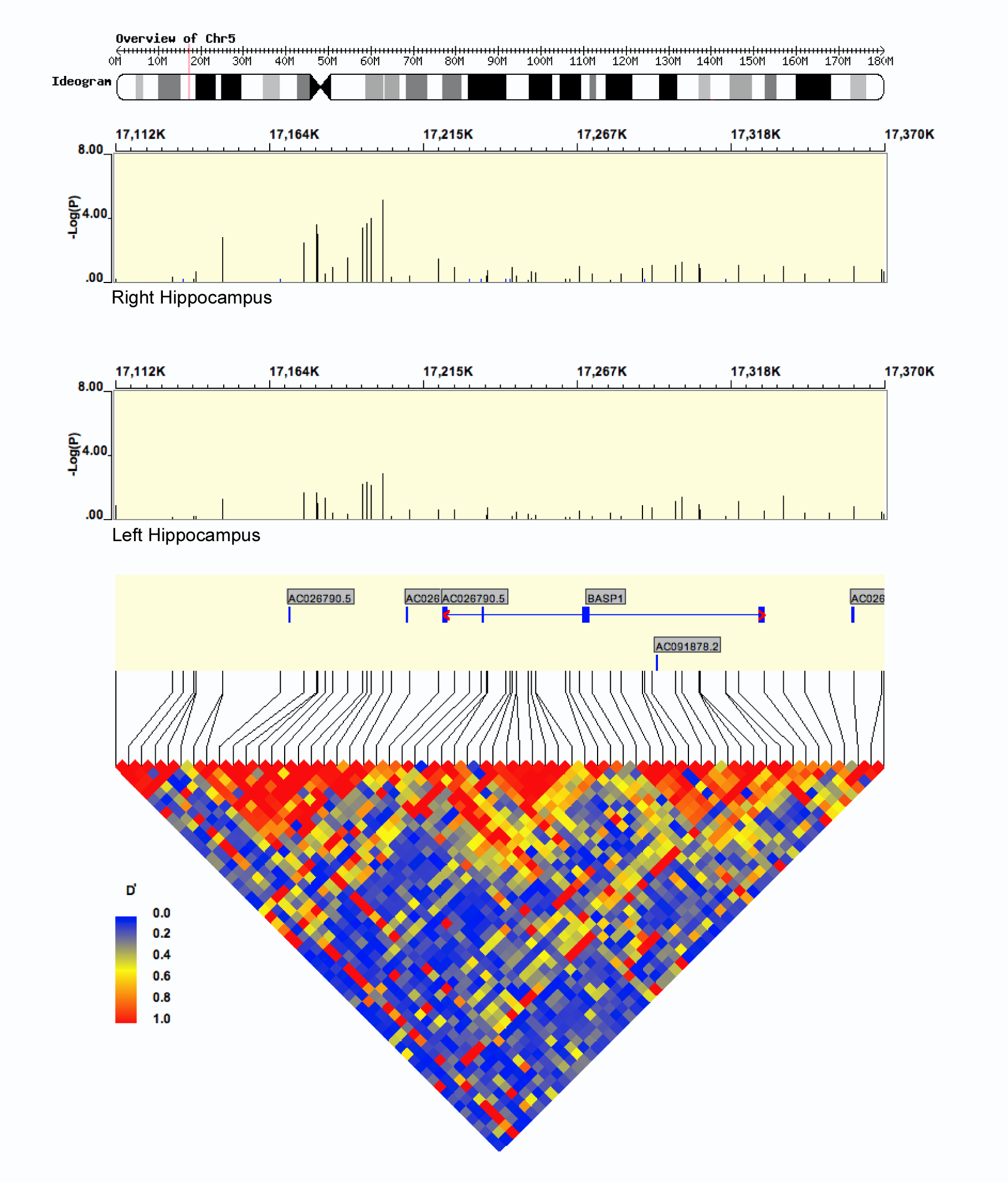

Supplement: Figure S2 — QT analysis of SNPs associated with genes or chromosomal regions as reported in Table 3 of the manuscript. Physical map of the SNPs associated with genes or chromosomal regions in the ADNI sample produced by WGAViewer. The top of the figure is the ideogram of the chromosome; the vertical red line depicts the relative location of locus of interest. Below the graph are the -log p significance values of the individual SNPs on the imaging phenotype (hippocampal atrophy) for the left and right hemispheres as indicated in each figure. The blue lines below the graph indicate the location of the exons in the transcripts annotated (translated region of the DNA). The vertical lines above the accompanying triangular matrix indicate the SNP locations, and demonstrate the LD pattern between SNPs (D'). The warmer colors on the flame scale indicate greater LD while the blue indicates absence of LD. (0.85 MB TIF) [file pone.0006501.s002.tif]

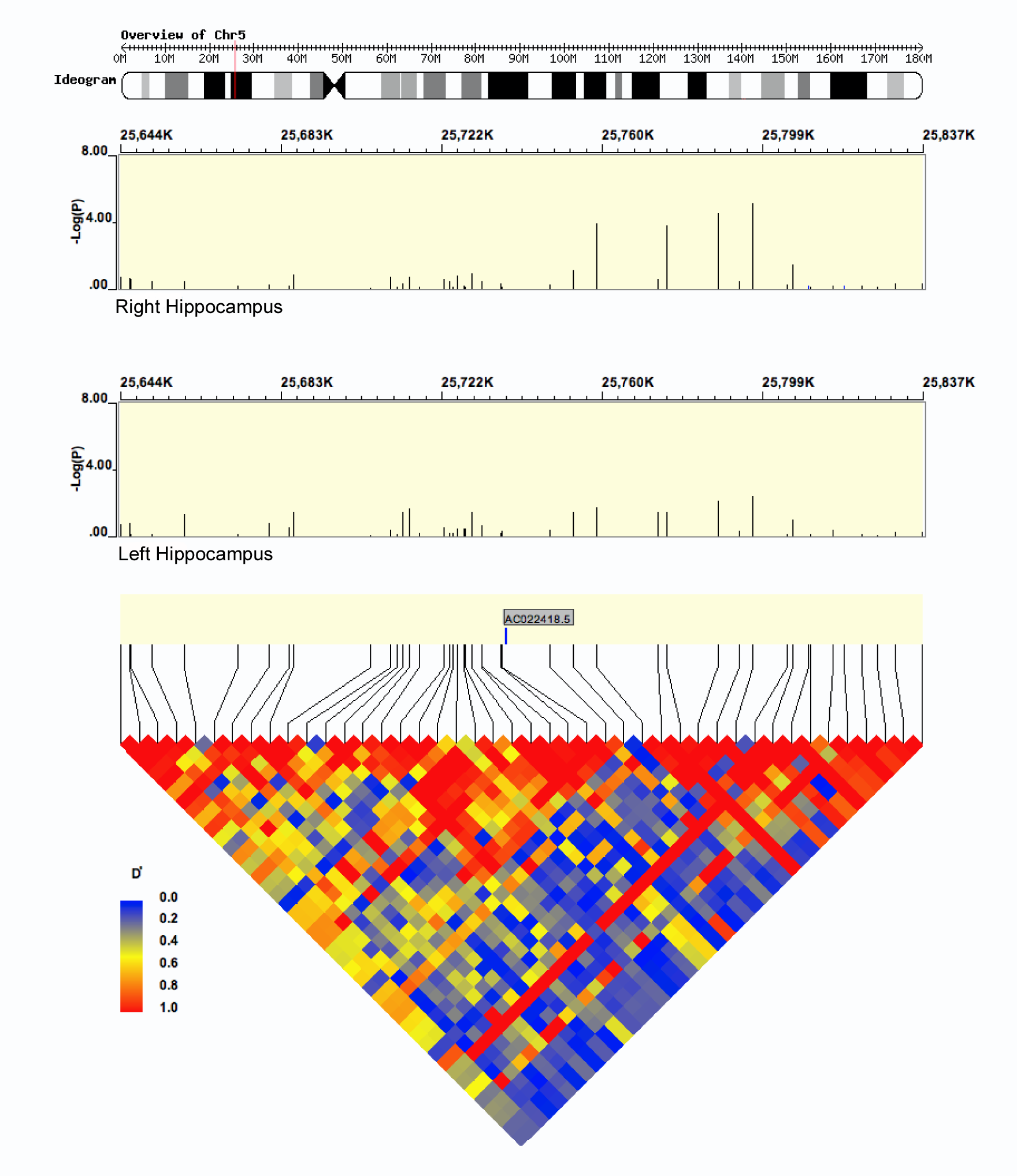

Supplement: Figure S3 — QT analysis of SNPs associated with genes or chromosomal regions as reported in Table 3 of the manuscript. Physical map of the SNPs associated with genes or chromosomal regions in the ADNI sample produced by WGAViewer. The top of the figure is the ideogram of the chromosome; the vertical red line depicts the relative location of locus of interest. Below the graph are the -log p significance values of the individual SNPs on the imaging phenotype (hippocampal atrophy) for the left and right hemispheres as indicated in each figure. The blue lines below the graph indicate the location of the exons in the transcripts annotated (translated region of the DNA). The vertical lines above the accompanying triangular matrix indicate the SNP locations, and demonstrate the LD pattern between SNPs (D'). The warmer colors on the flame scale indicate greater LD while the blue indicates absence of LD. (0.71 MB TIF) [file pone.0006501.s003.tif]

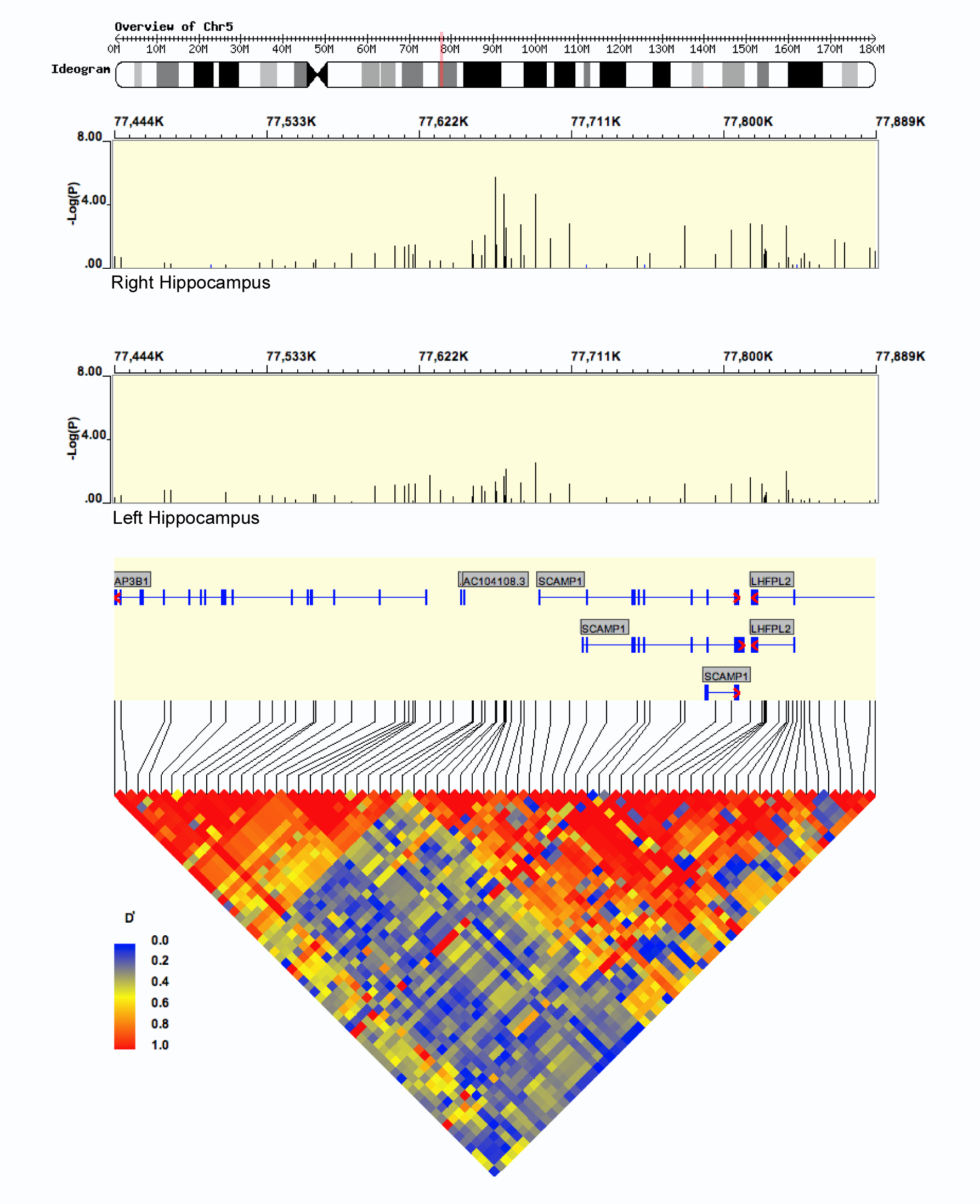

Supplement: Figure S4 — QT analysis of SNPs associated with genes or chromosomal regions as reported in Table 3 of the manuscript. Physical map of the SNPs associated with genes or chromosomal regions in the ADNI sample produced by WGAViewer. The top of the figure is the ideogram of the chromosome; the vertical red line depicts the relative location of locus of interest. Below the graph are the -log p significance values of the individual SNPs on the imaging phenotype (hippocampal atrophy) for the left and right hemispheres as indicated in each figure. The blue lines below the graph indicate the location of the exons in the transcripts annotated (translated region of the DNA). The vertical lines above the accompanying triangular matrix indicate the SNP locations, and demonstrate the LD pattern between SNPs (D'). The warmer colors on the flame scale indicate greater LD while the blue indicates absence of LD. (0.87 MB TIF) [file pone.0006501.s004.tif]

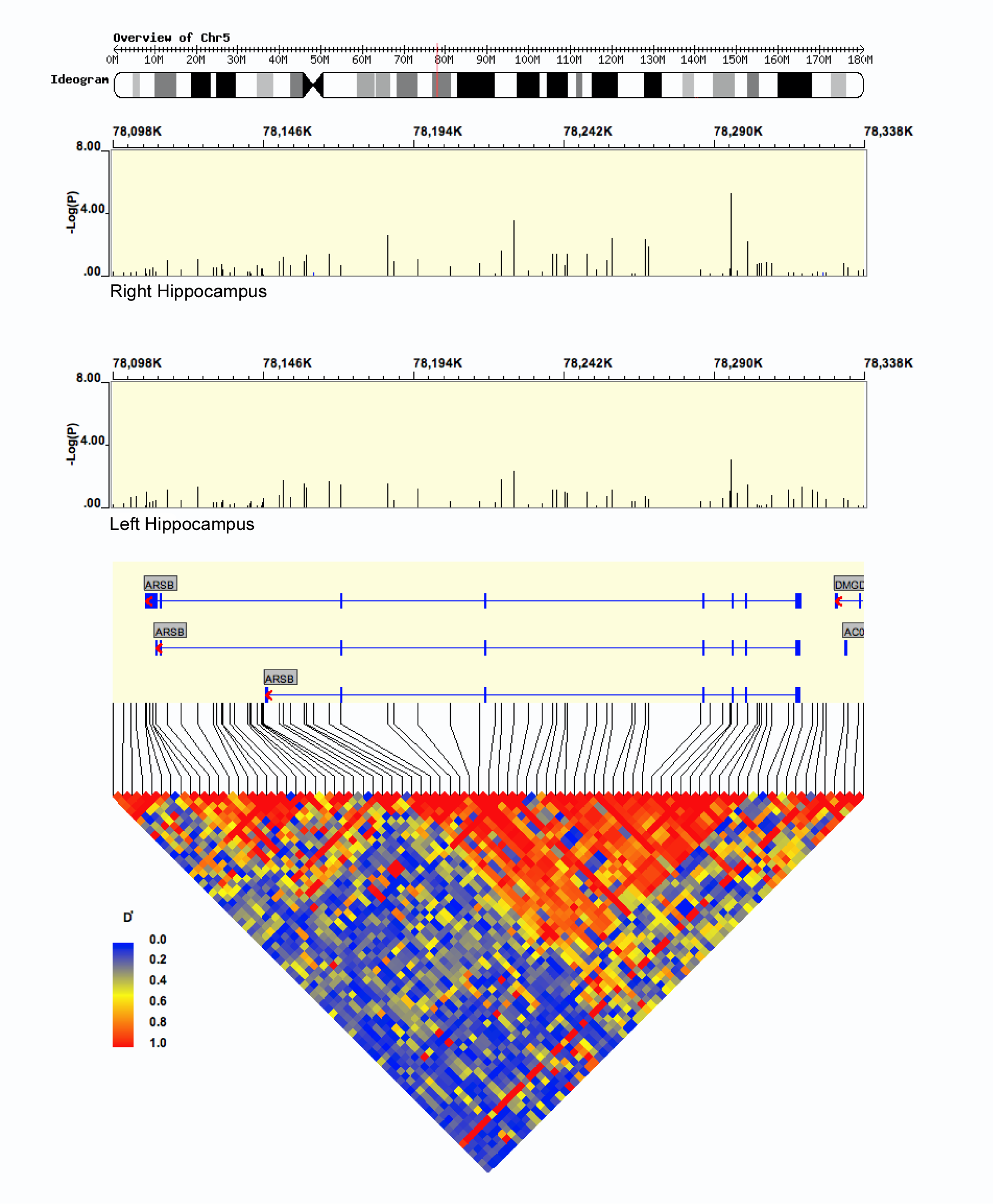

Supplement: Figure S5 — QT analysis of SNPs associated with genes or chromosomal regions as reported in Table 3 of the manuscript. Physical map of the SNPs associated with genes or chromosomal regions in the ADNI sample produced by WGAViewer. The top of the figure is the ideogram of the chromosome; the vertical red line depicts the relative location of locus of interest. Below the graph are the -log p significance values of the individual SNPs on the imaging phenotype (hippocampal atrophy) for the left and right hemispheres as indicated in each figure. The blue lines below the graph indicate the location of the exons in the transcripts annotated (translated region of the DNA). The vertical lines above the accompanying triangular matrix indicate the SNP locations, and demonstrate the LD pattern between SNPs (D'). The warmer colors on the flame scale indicate greater LD while the blue indicates absence of LD. (0.95 MB TIF) [file pone.0006501.s005.tif]

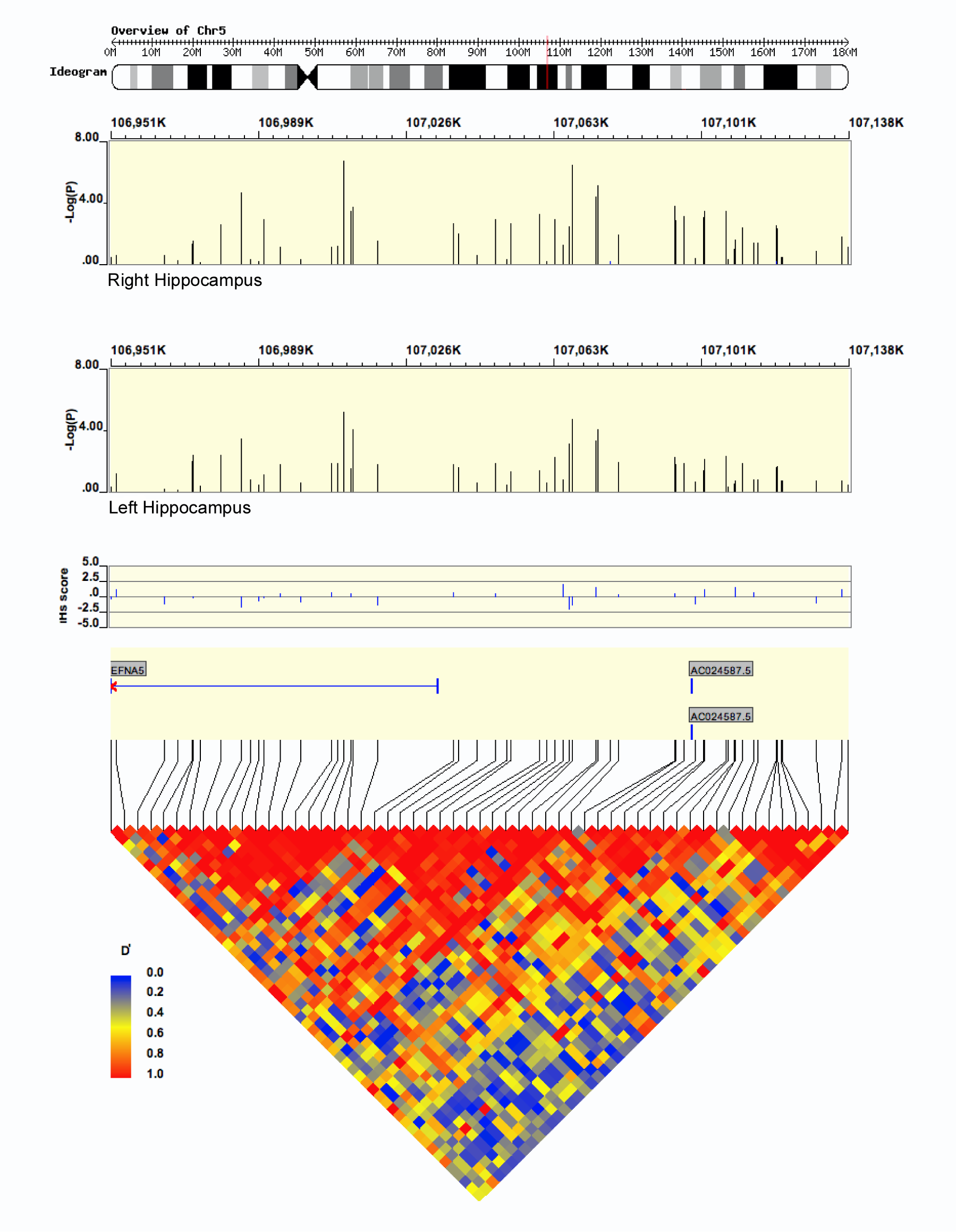

Supplement: Figure S6 — QT analysis of SNPs associated with genes or chromosomal regions as reported in Table 3 of the manuscript. Physical map of the SNPs associated with genes or chromosomal regions in the ADNI sample produced by WGAViewer. The top of the figure is the ideogram of the chromosome; the vertical red line depicts the relative location of locus of interest. Below the graph are the -log p significance values of the individual SNPs on the imaging phenotype (hippocampal atrophy) for the left and right hemispheres as indicated in each figure. The blue lines below the graph indicate the location of the exons in the transcripts annotated (translated region of the DNA). The vertical lines above the accompanying triangular matrix indicate the SNP locations, and demonstrate the LD pattern between SNPs (D'). The warmer colors on the flame scale indicate greater LD while the blue indicates absence of LD. (0.85 MB TIF) [file pone.0006501.s006.tif]

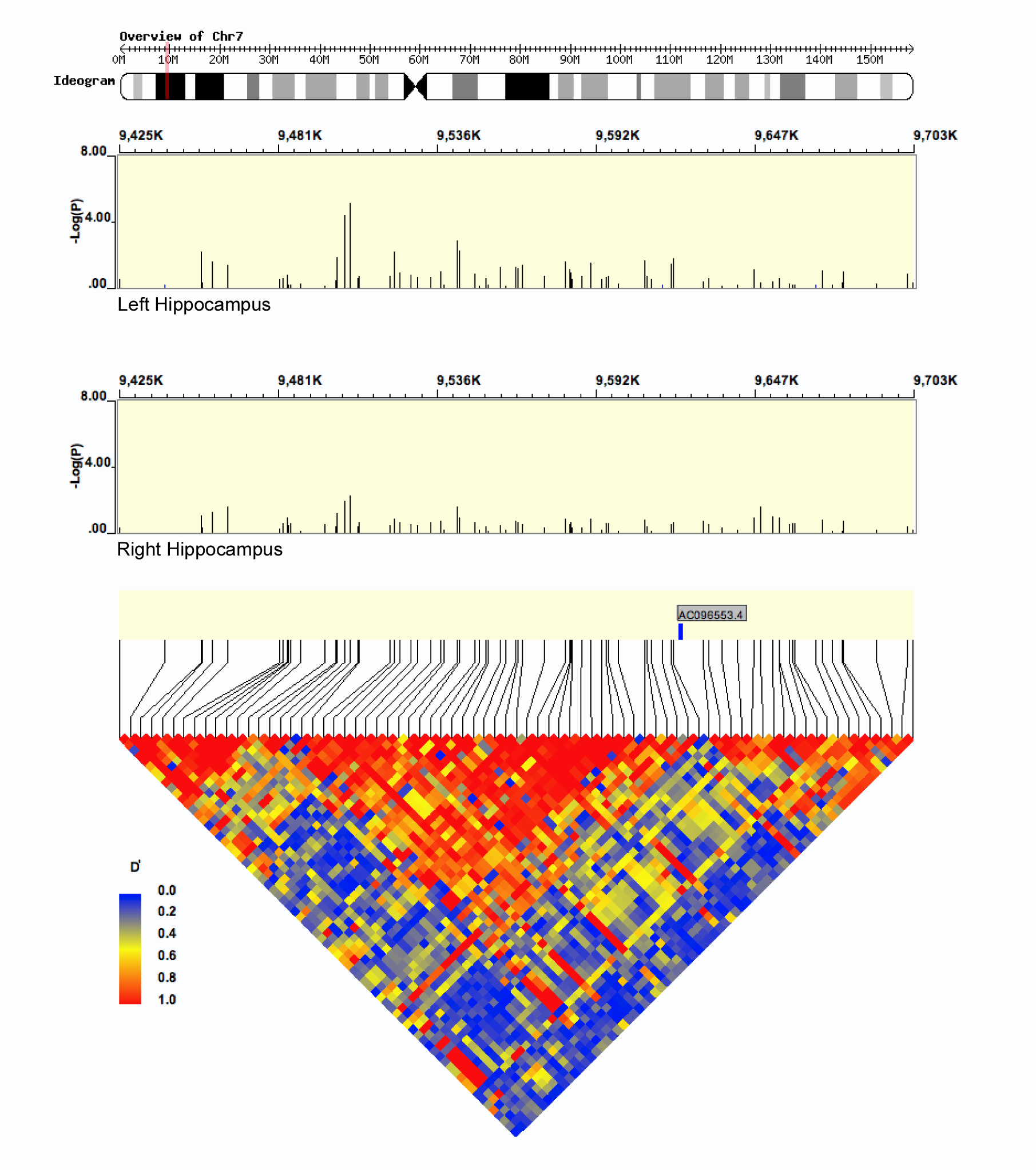

Supplement: Figure S7 — QT analysis of SNPs associated with genes or chromosomal regions as reported in Table 3 of the manuscript. Physical map of the SNPs associated with genes or chromosomal regions in the ADNI sample produced by WGAViewer. The top of the figure is the ideogram of the chromosome; the vertical red line depicts the relative location of locus of interest. Below the graph are the -log p significance values of the individual SNPs on the imaging phenotype (hippocampal atrophy) for the left and right hemispheres as indicated in each figure. The blue lines below the graph indicate the location of the exons in the transcripts annotated (translated region of the DNA). The vertical lines above the accompanying triangular matrix indicate the SNP locations, and demonstrate the LD pattern between SNPs (D'). The warmer colors on the flame scale indicate greater LD while the blue indicates absence of LD. (0.87 MB TIF) [file pone.0006501.s007.tif]

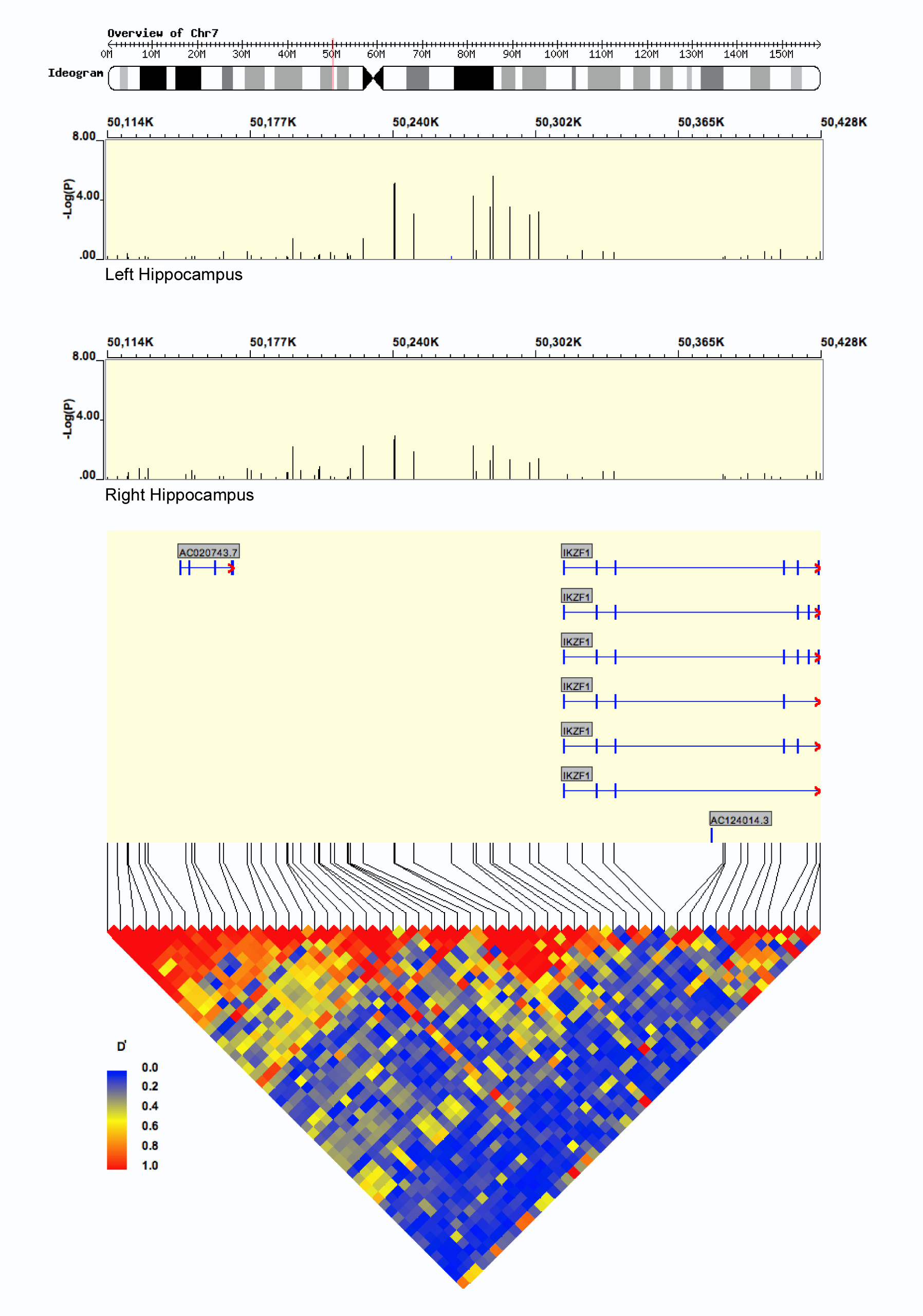

Supplement: Figure S8 — QT analysis of SNPs associated with genes or chromosomal regions as reported in Table 3 of the manuscript. Physical map of the SNPs associated with genes or chromosomal regions in the ADNI sample produced by WGAViewer. The top of the figure is the ideogram of the chromosome; the vertical red line depicts the relative location of locus of interest. Below the graph are the -log p significance values of the individual SNPs on the imaging phenotype (hippocampal atrophy) for the left and right hemispheres as indicated in each figure. The blue lines below the graph indicate the location of the exons in the transcripts annotated (translated region of the DNA). The vertical lines above the accompanying triangular matrix indicate the SNP locations, and demonstrate the LD pattern between SNPs (D'). The warmer colors on the flame scale indicate greater LD while the blue indicates absence of LD. (0.90 MB TIF) [file pone.0006501.s008.tif]

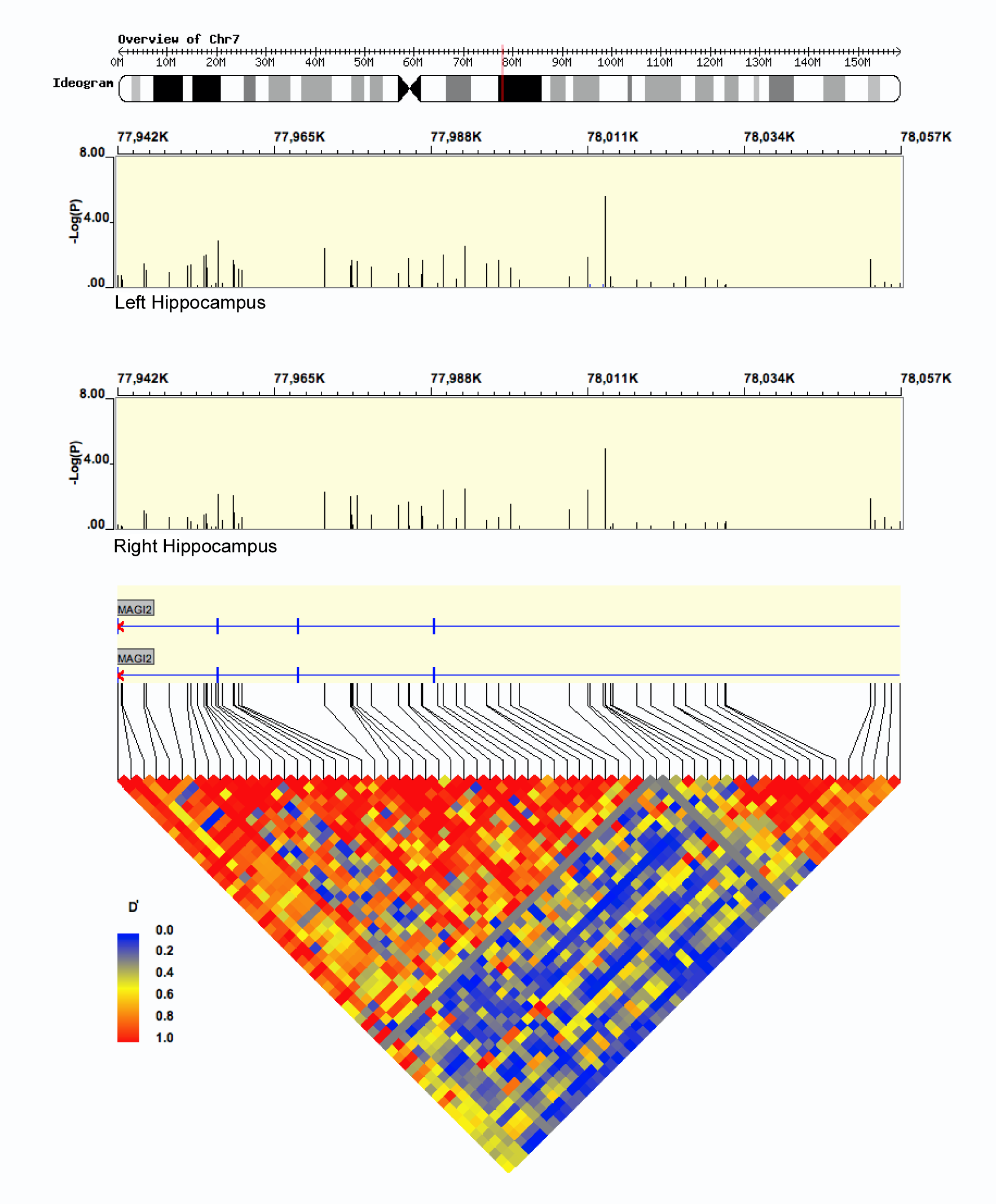

Supplement: Figure S9 — QT analysis of SNPs associated with genes or chromosomal regions as reported in Table 3 of the manuscript. Physical map of the SNPs associated with genes or chromosomal regions in the ADNI sample produced by WGAViewer. The top of the figure is the ideogram of the chromosome; the vertical red line depicts the relative location of locus of interest. Below the graph are the -log p significance values of the individual SNPs on the imaging phenotype (hippocampal atrophy) for the left and right hemispheres as indicated in each figure. The blue lines below the graph indicate the location of the exons in the transcripts annotated (translated region of the DNA). The vertical lines above the accompanying triangular matrix indicate the SNP locations, and demonstrate the LD pattern between SNPs (D'). The warmer colors on the flame scale indicate greater LD while the blue indicates absence of LD. (0.82 MB TIF) [file pone.0006501.s009.tif]

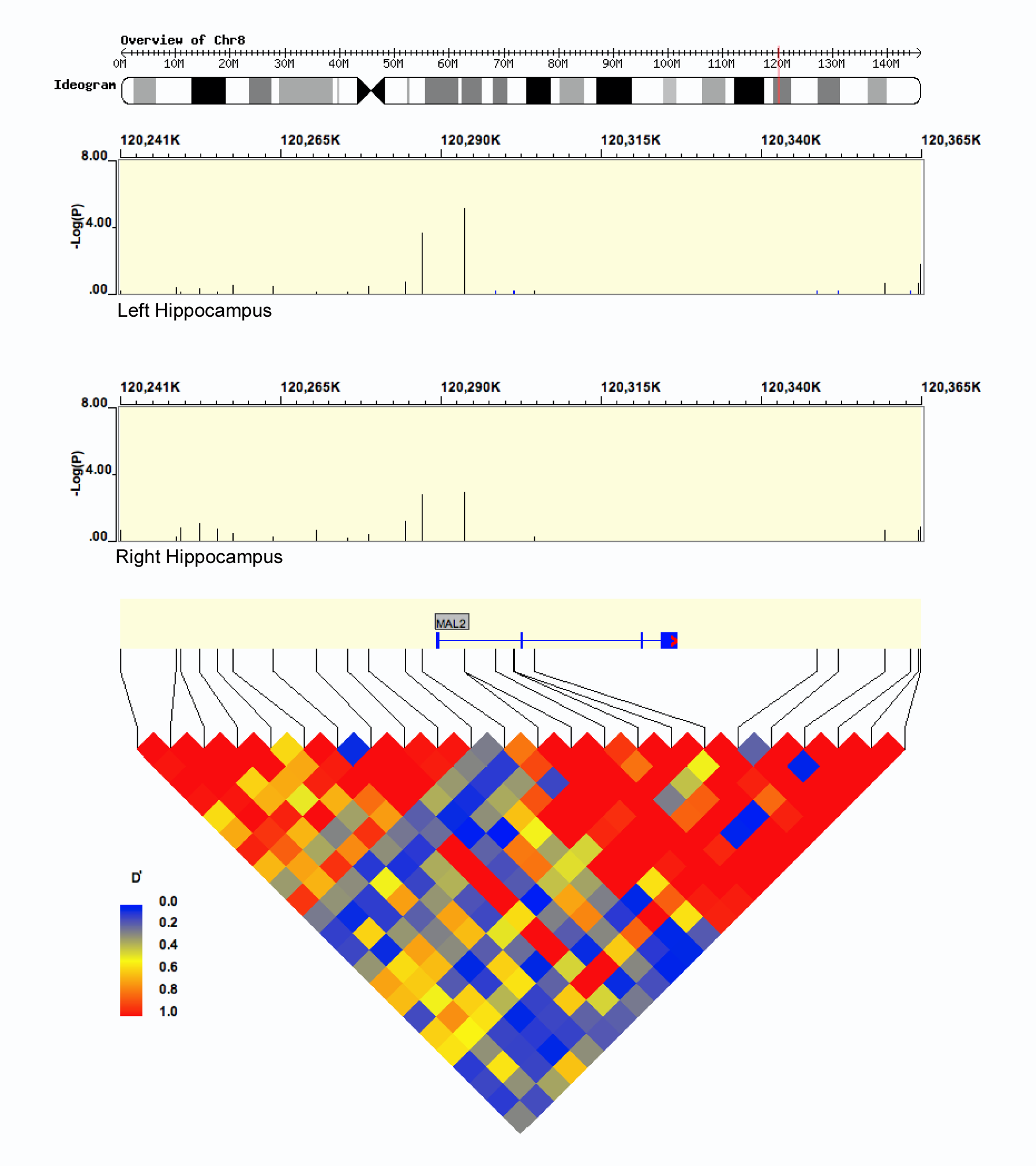

Supplement: Figure S10 — QT analysis of SNPs associated with genes or chromosomal regions as reported in Table 3 of the manuscript. Physical map of the SNPs associated with genes or chromosomal regions in the ADNI sample produced by WGAViewer. The top of the figure is the ideogram of the chromosome; the vertical red line depicts the relative location of locus of interest. Below the graph are the -log p significance values of the individual SNPs on the imaging phenotype (hippocampal atrophy) for the left and right hemispheres as indicated in each figure. The blue lines below the graph indicate the location of the exons in the transcripts annotated (translated region of the DNA). The vertical lines above the accompanying triangular matrix indicate the SNP locations, and demonstrate the LD pattern between SNPs (D'). The warmer colors on the flame scale indicate greater LD while the blue indicates absence of LD. (0.57 MB TIF) [file pone.0006501.s010.tif]

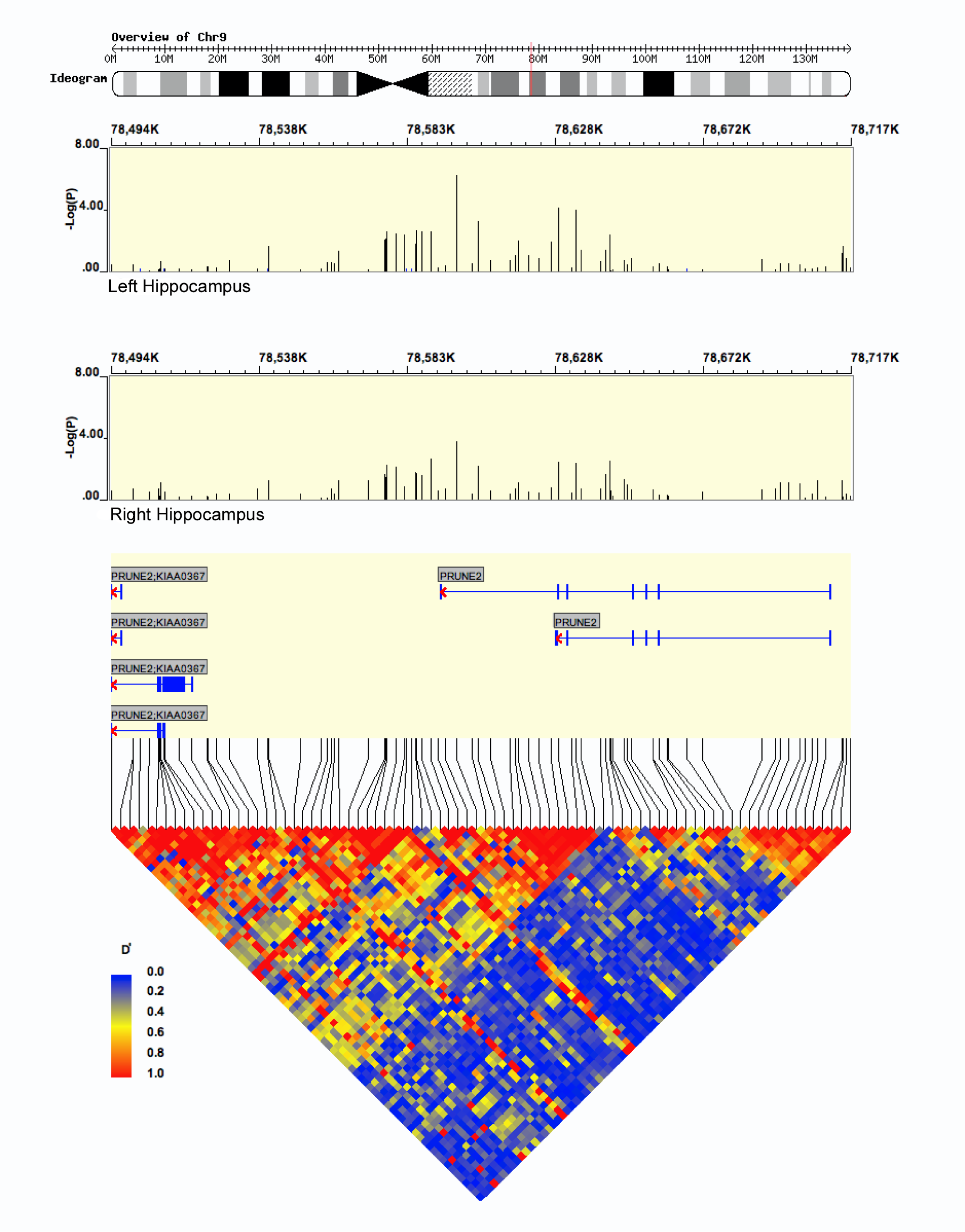

Supplement: Figure S11 — QT analysis of SNPs associated with genes or chromosomal regions as reported in Table 3 of the manuscript. Physical map of the SNPs associated with genes or chromosomal regions in the ADNI sample produced by WGAViewer. The top of the figure is the ideogram of the chromosome; the vertical red line depicts the relative location of locus of interest. Below the graph are the -log p significance values of the individual SNPs on the imaging phenotype (hippocampal atrophy) for the left and right hemispheres as indicated in each figure. The blue lines below the graph indicate the location of the exons in the transcripts annotated (translated region of the DNA). The vertical lines above the accompanying triangular matrix indicate the SNP locations, and demonstrate the LD pattern between SNPs (D'). The warmer colors on the flame scale indicate greater LD while the blue indicates absence of LD. (1.01 MB TIF) [file pone.0006501.s011.tif]

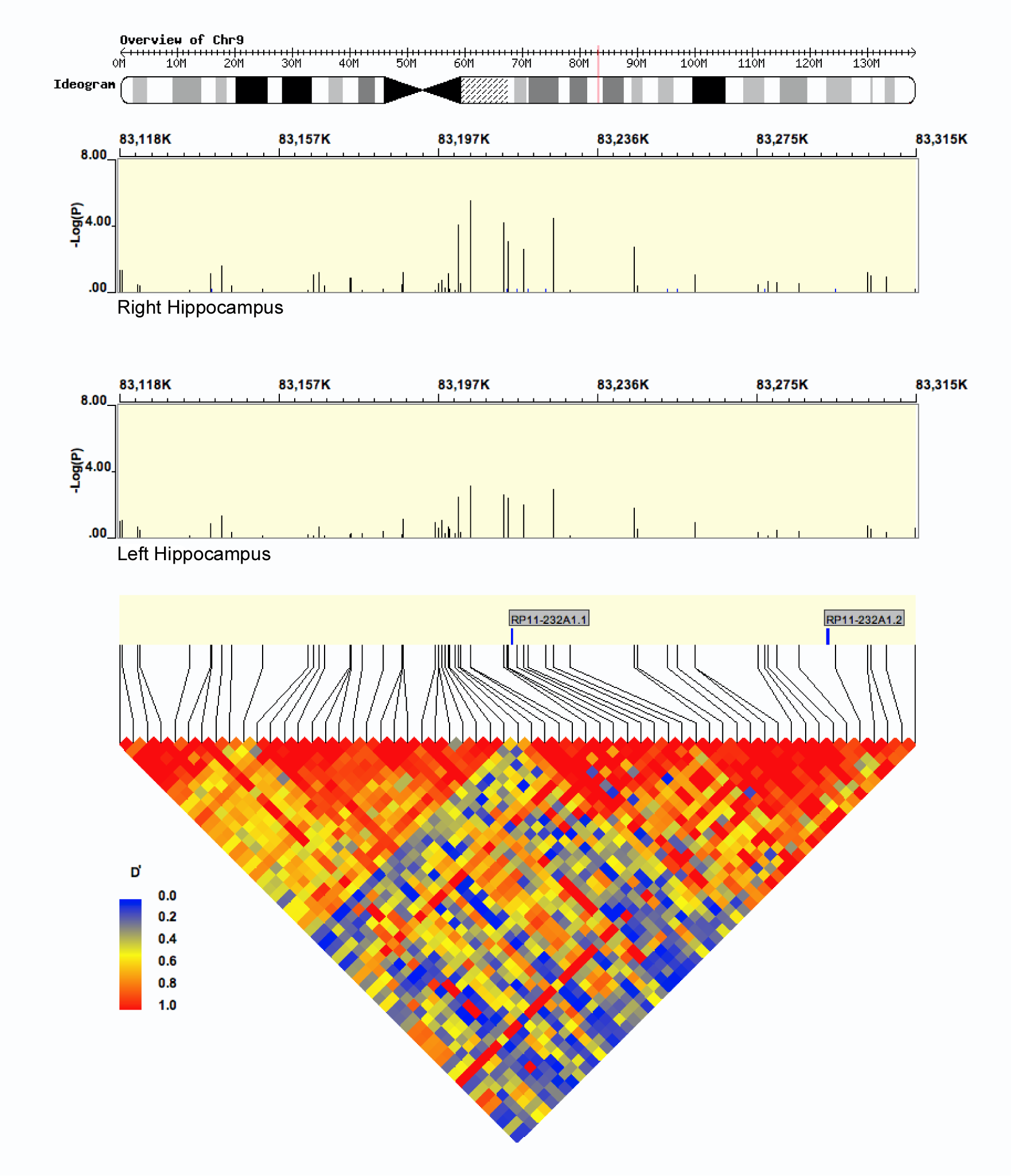

Supplement: Figure S12 — QT analysis of SNPs associated with genes or chromosomal regions as reported in Table 3 of the manuscript. Physical map of the SNPs associated with genes or chromosomal regions in the ADNI sample produced by WGAViewer. The top of the figure is the ideogram of the chromosome; the vertical red line depicts the relative location of locus of interest. Below the graph are the -log p significance values of the individual SNPs on the imaging phenotype (hippocampal atrophy) for the left and right hemispheres as indicated in each figure. The blue lines below the graph indicate the location of the exons in the transcripts annotated (translated region of the DNA). The vertical lines above the accompanying triangular matrix indicate the SNP locations, and demonstrate the LD pattern between SNPs (D'). The warmer colors on the flame scale indicate greater LD while the blue indicates absence of LD. (0.78 MB TIF) [file pone.0006501.s012.tif]

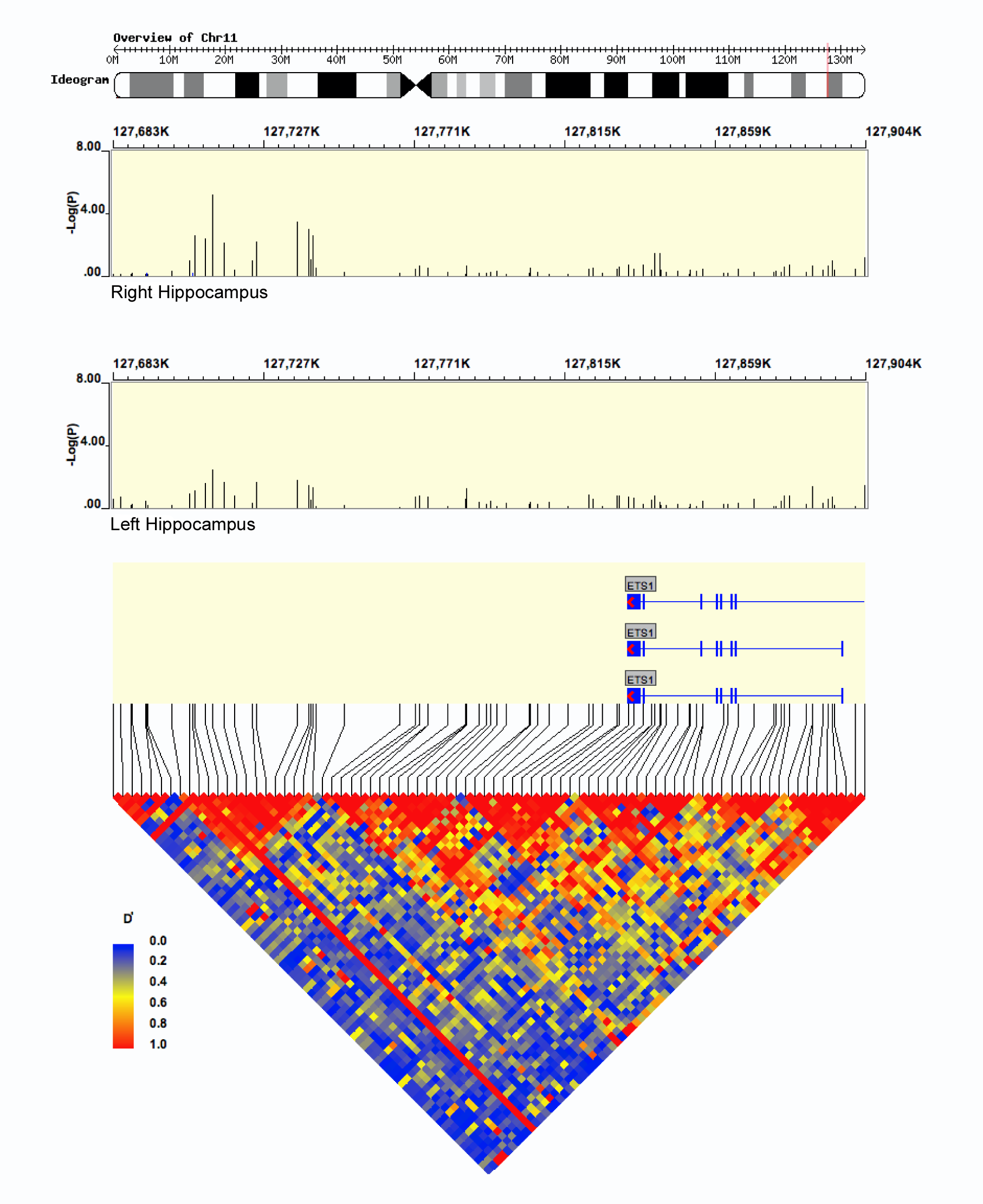

Supplement: Figure S13 — QT analysis of SNPs associated with genes or chromosomal regions as reported in Table 3 of the manuscript. Physical map of the SNPs associated with genes or chromosomal regions in the ADNI sample produced by WGAViewer. The top of the figure is the ideogram of the chromosome; the vertical red line depicts the relative location of locus of interest. Below the graph are the -log p significance values of the individual SNPs on the imaging phenotype (hippocampal atrophy) for the left and right hemispheres as indicated in each figure. The blue lines below the graph indicate the location of the exons in the transcripts annotated (translated region of the DNA). The vertical lines above the accompanying triangular matrix indicate the SNP locations, and demonstrate the LD pattern between SNPs (D'). The warmer colors on the flame scale indicate greater LD while the blue indicates absence of LD. (0.95 MB TIF) [file pone.0006501.s013.tif]

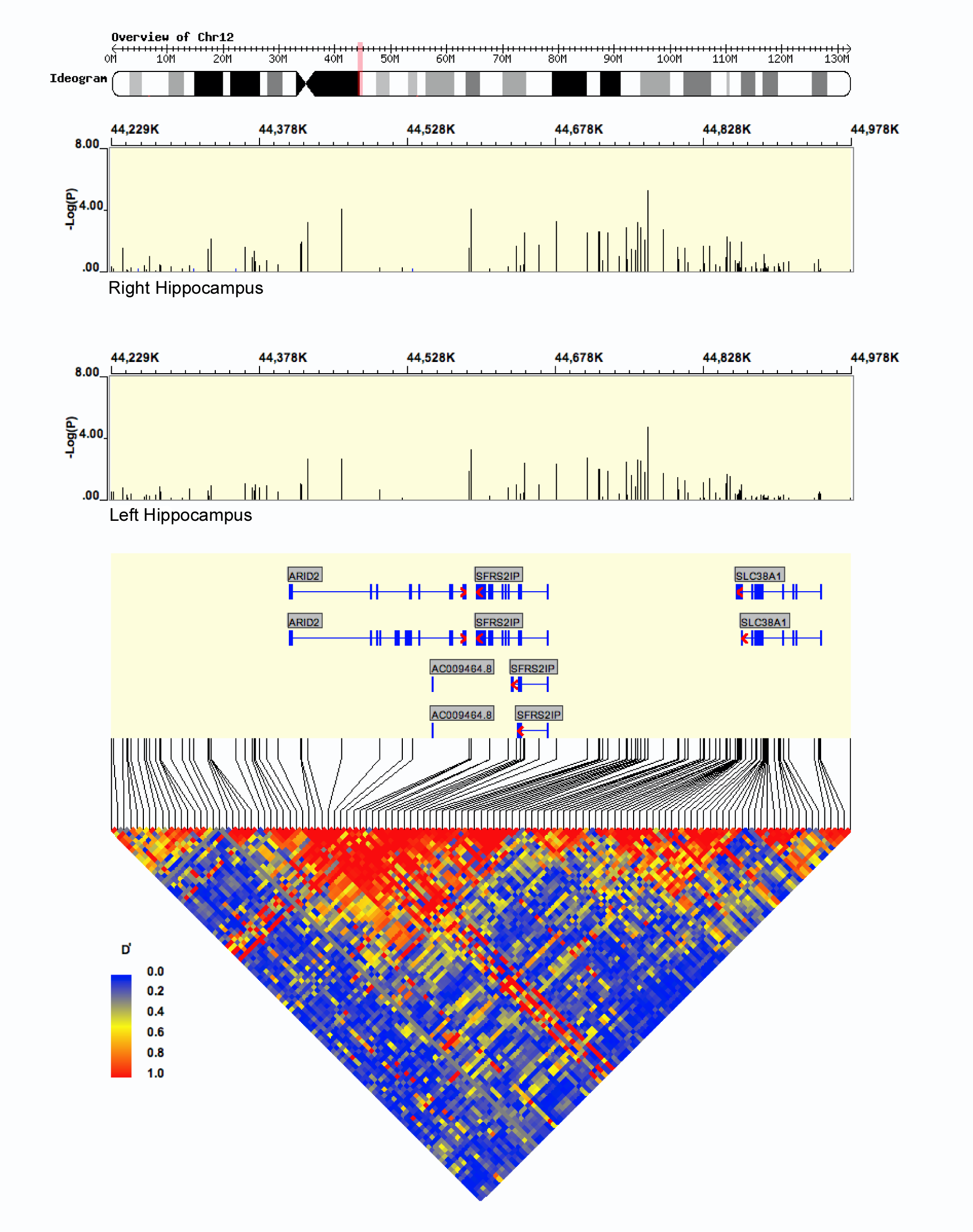

Supplement: Figure S14 — QT analysis of SNPs associated with genes or chromosomal regions as reported in Table 3 of the manuscript. Physical map of the SNPs associated with genes or chromosomal regions in the ADNI sample produced by WGAViewer. The top of the figure is the ideogram of the chromosome; the vertical red line depicts the relative location of locus of interest. Below the graph are the -log p significance values of the individual SNPs on the imaging phenotype (hippocampal atrophy) for the left and right hemispheres as indicated in each figure. The blue lines below the graph indicate the location of the exons in the transcripts annotated (translated region of the DNA). The vertical lines above the accompanying triangular matrix indicate the SNP locations, and demonstrate the LD pattern between SNPs (D'). The warmer colors on the flame scale indicate greater LD while the blue indicates absence of LD. (1.20 MB TIF) [file pone.0006501.s014.tif]

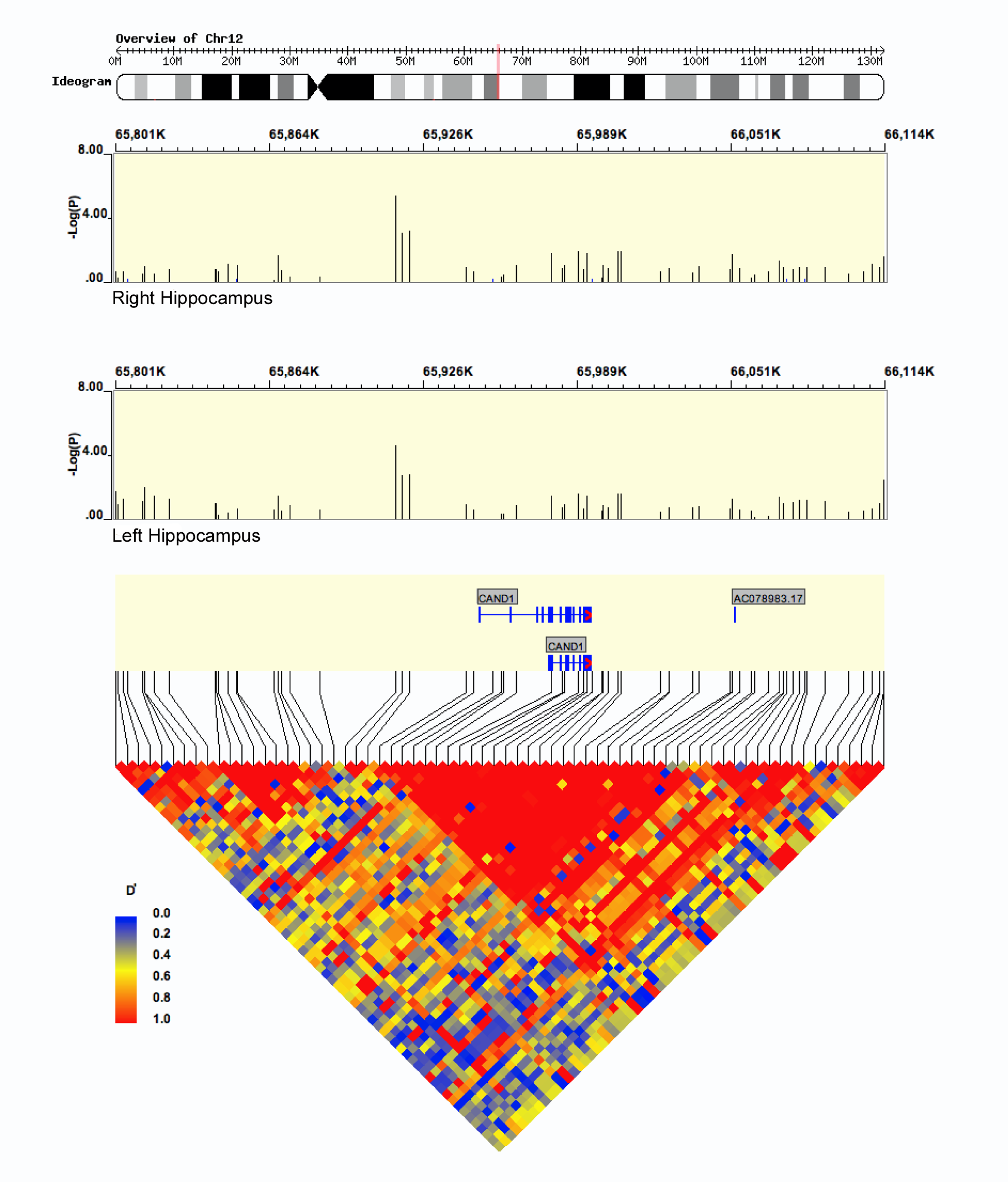

Supplement: Figure S15 — QT analysis of SNPs associated with genes or chromosomal regions as reported in Table 3 of the manuscript. Physical map of the SNPs associated with genes or chromosomal regions in the ADNI sample produced by WGAViewer. The top of the figure is the ideogram of the chromosome; the vertical red line depicts the relative location of locus of interest. Below the graph are the -log p significance values of the individual SNPs on the imaging phenotype (hippocampal atrophy) for the left and right hemispheres as indicated in each figure. The blue lines below the graph indicate the location of the exons in the transcripts annotated (translated region of the DNA). The vertical lines above the accompanying triangular matrix indicate the SNP locations, and demonstrate the LD pattern between SNPs (D'). The warmer colors on the flame scale indicate greater LD while the blue indicates absence of LD. (0.82 MB TIF) [file pone.0006501.s015.tif]

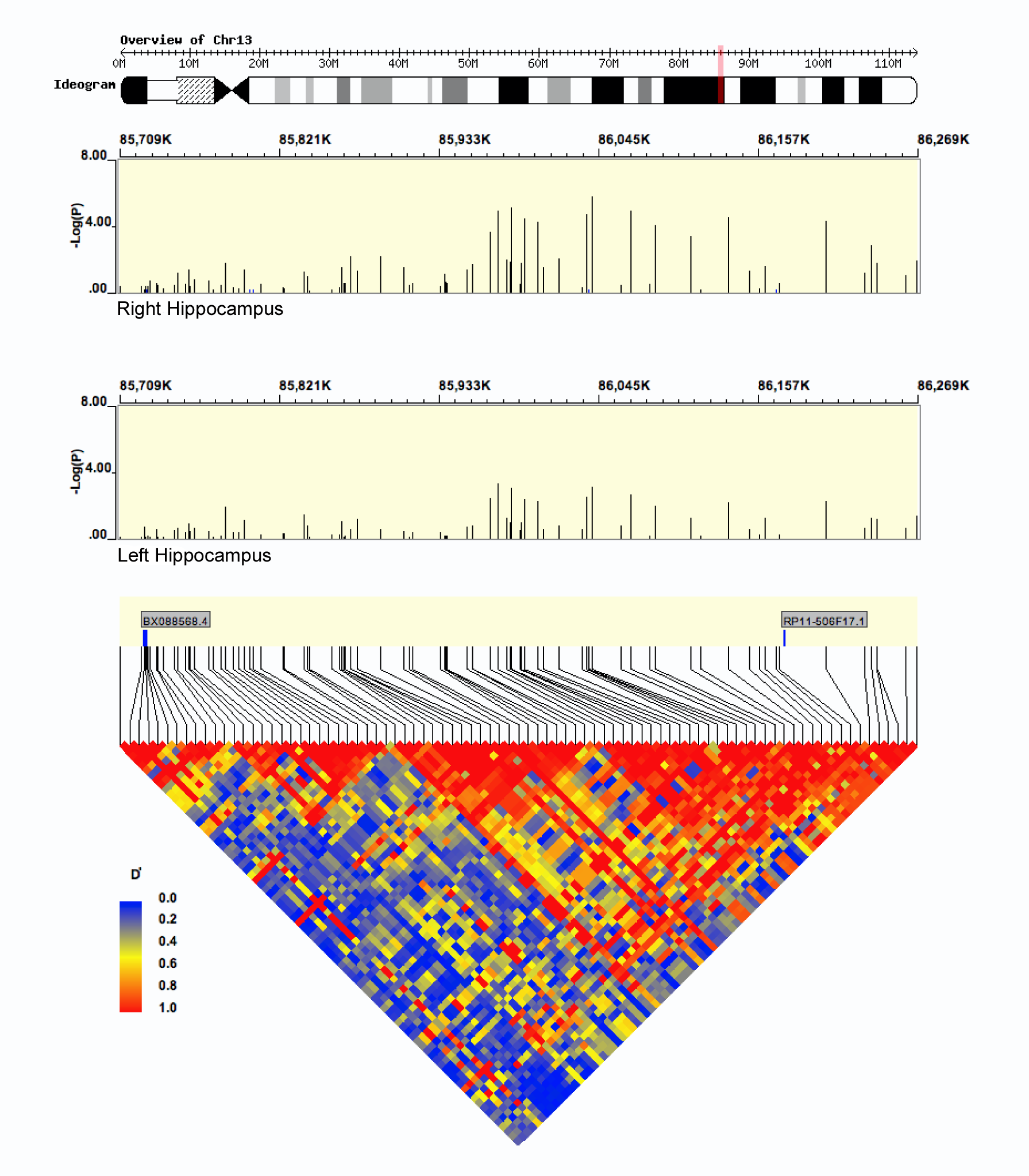

Supplement: Figure S16 — QT analysis of SNPs associated with genes or chromosomal regions as reported in Table 3 of the manuscript. Physical map of the SNPs associated with genes or chromosomal regions in the ADNI sample produced by WGAViewer. The top of the figure is the ideogram of the chromosome; the vertical red line depicts the relative location of locus of interest. Below the graph are the -log p significance values of the individual SNPs on the imaging phenotype (hippocampal atrophy) for the left and right hemispheres as indicated in each figure. The blue lines below the graph indicate the location of the exons in the transcripts annotated (translated region of the DNA). The vertical lines above the accompanying triangular matrix indicate the SNP locations, and demonstrate the LD pattern between SNPs (D'). The warmer colors on the flame scale indicate greater LD while the blue indicates absence of LD. (0.93 MB TIF) [file pone.0006501.s016.tif]

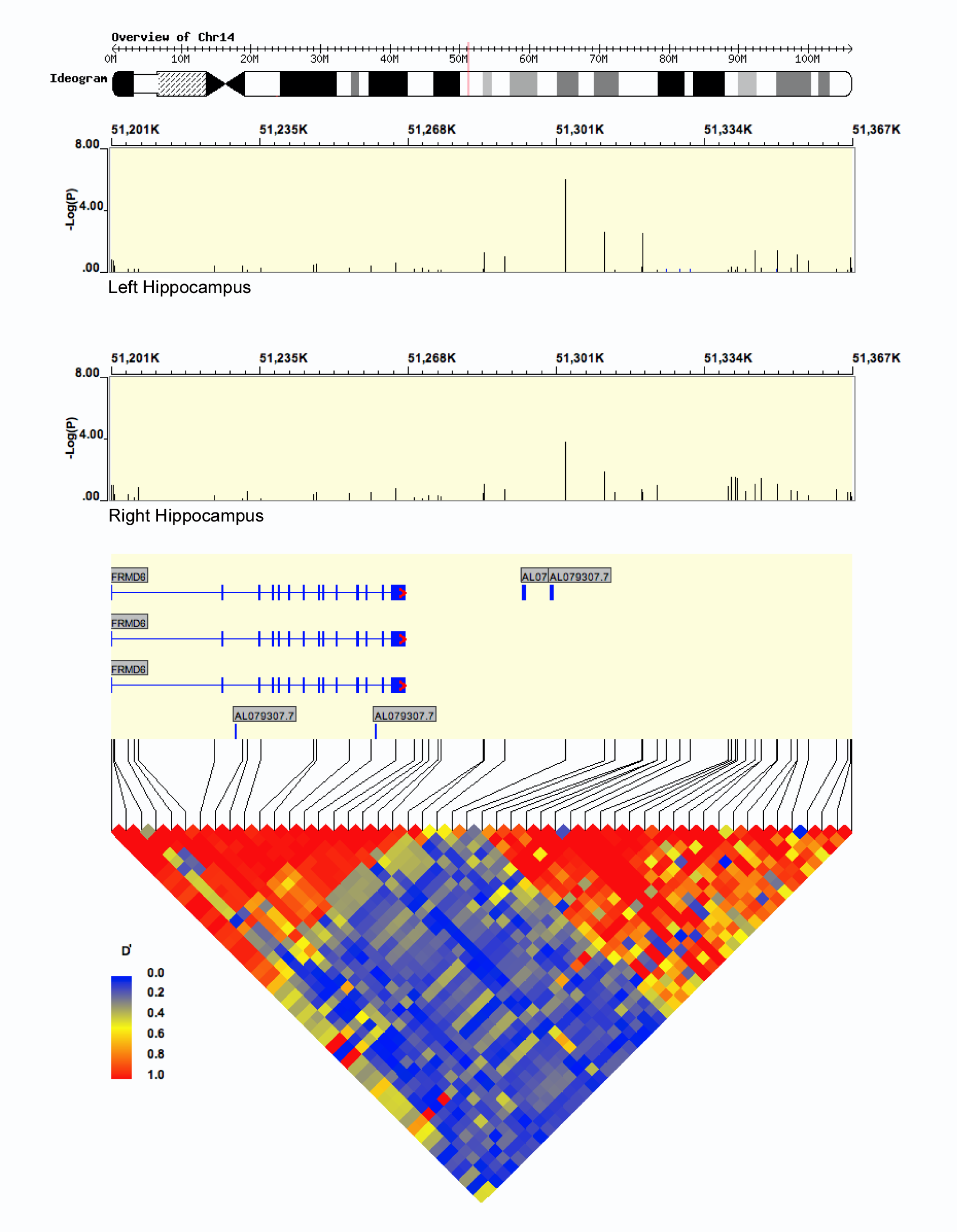

Supplement: Figure S17 — QT analysis of SNPs associated with genes or chromosomal regions as reported in Table 3 of the manuscript. Physical map of the SNPs associated with genes or chromosomal regions in the ADNI sample produced by WGAViewer. The top of the figure is the ideogram of the chromosome; the vertical red line depicts the relative location of locus of interest. Below the graph are the -log p significance values of the individual SNPs on the imaging phenotype (hippocampal atrophy) for the left and right hemispheres as indicated in each figure. The blue lines below the graph indicate the location of the exons in the transcripts annotated (translated region of the DNA). The vertical lines above the accompanying triangular matrix indicate the SNP locations, and demonstrate the LD pattern between SNPs (D'). The warmer colors on the flame scale indicate greater LD while the blue indicates absence of LD. (0.79 MB TIF) [file pone.0006501.s017.tif]

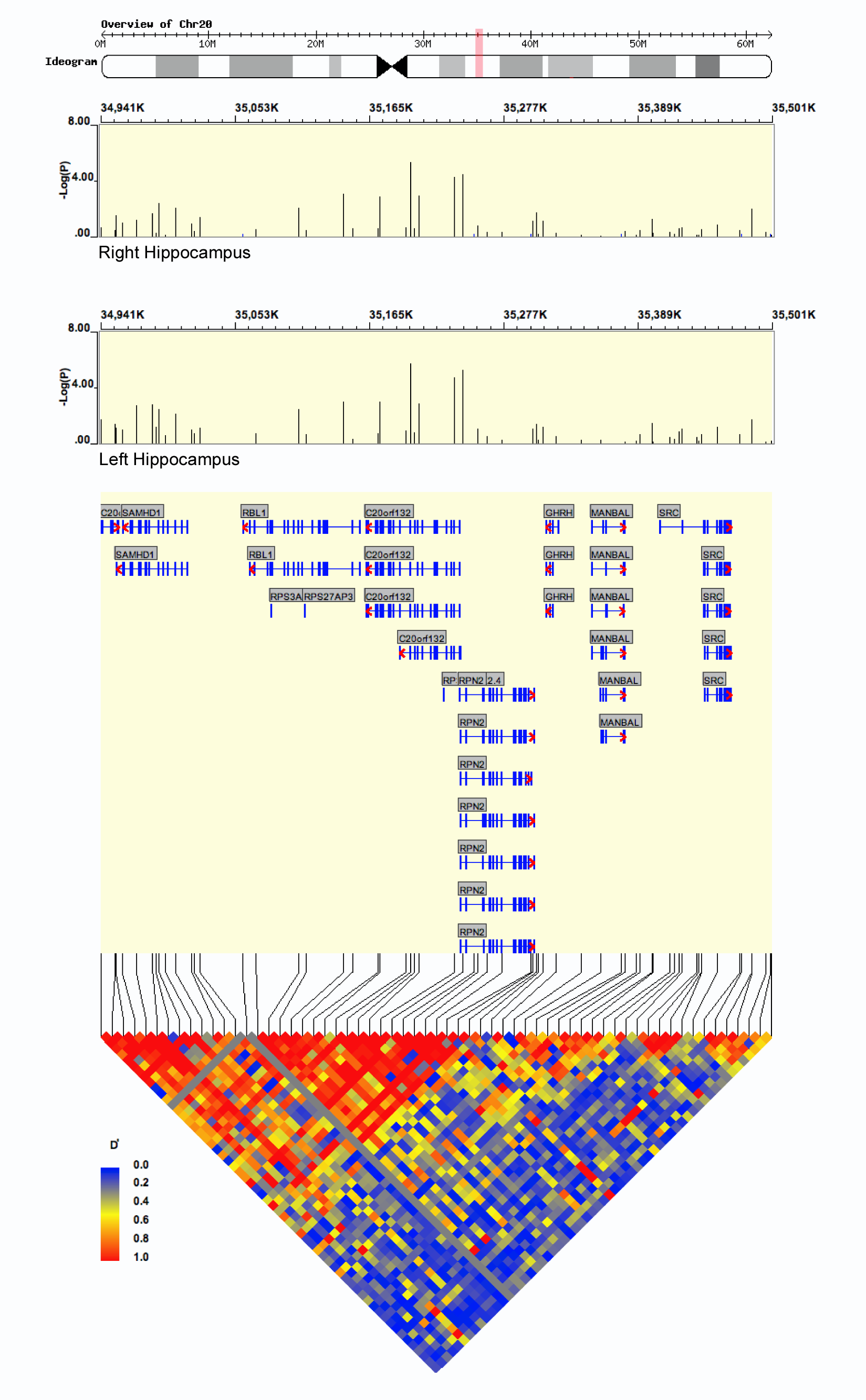

Supplement: Figure S18 — QT analysis of SNPs associated with genes or chromosomal regions as reported in Table 3 of the manuscript. Physical map of the SNPs associated with genes or chromosomal regions in the ADNI sample produced by WGAViewer. The top of the figure is the ideogram of the chromosome; the vertical red line depicts the relative location of locus of interest. Below the graph are the -log p significance values of the individual SNPs on the imaging phenotype (hippocampal atrophy) for the left and right hemispheres as indicated in each figure. The blue lines below the graph indicate the location of the exons in the transcripts annotated (translated region of the DNA). The vertical lines above the accompanying triangular matrix indicate the SNP locations, and demonstrate the LD pattern between SNPs (D'). The warmer colors on the flame scale indicate greater LD while the blue indicates absence of LD. (0.98 MB TIF) [file pone.0006501.s018.tif]

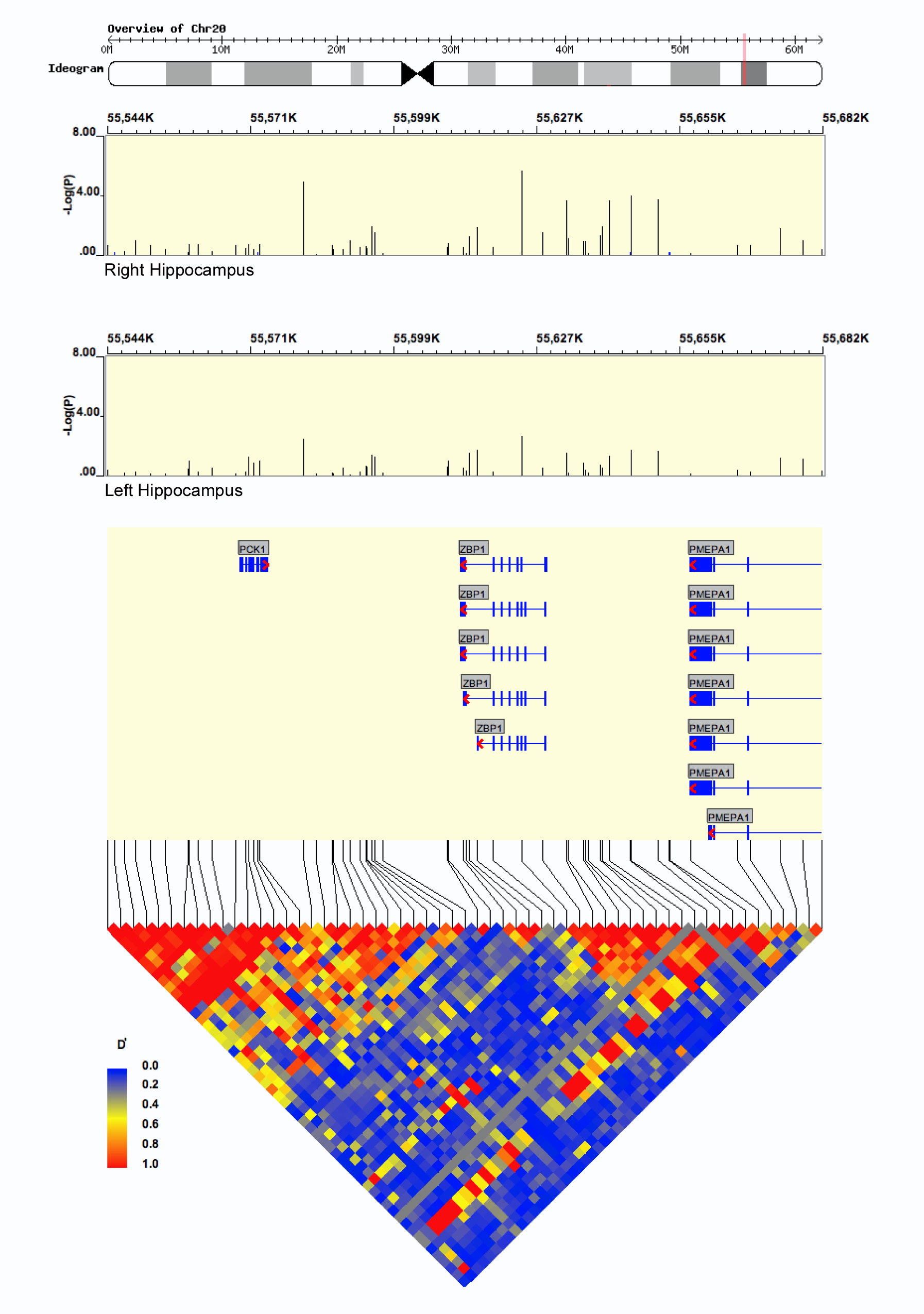

Supplement: Figure S19 — QT analysis of SNPs associated with genes or chromosomal regions as reported in Table 3 of the manuscript. Physical map of the SNPs associated with genes or chromosomal regions in the ADNI sample produced by WGAViewer. The top of the figure is the ideogram of the chromosome; the vertical red line depicts the relative location of locus of interest. Below the graph are the -log p significance values of the individual SNPs on the imaging phenotype (hippocampal atrophy) for the left and right hemispheres as indicated in each figure. The blue lines below the graph indicate the location of the exons in the transcripts annotated (translated region of the DNA). The vertical lines above the accompanying triangular matrix indicate the SNP locations, and demonstrate the LD pattern between SNPs (D'). The warmer colors on the flame scale indicate greater LD while the blue indicates absence of LD. (0.90 MB TIF) [file pone.0006501.s019.tif]

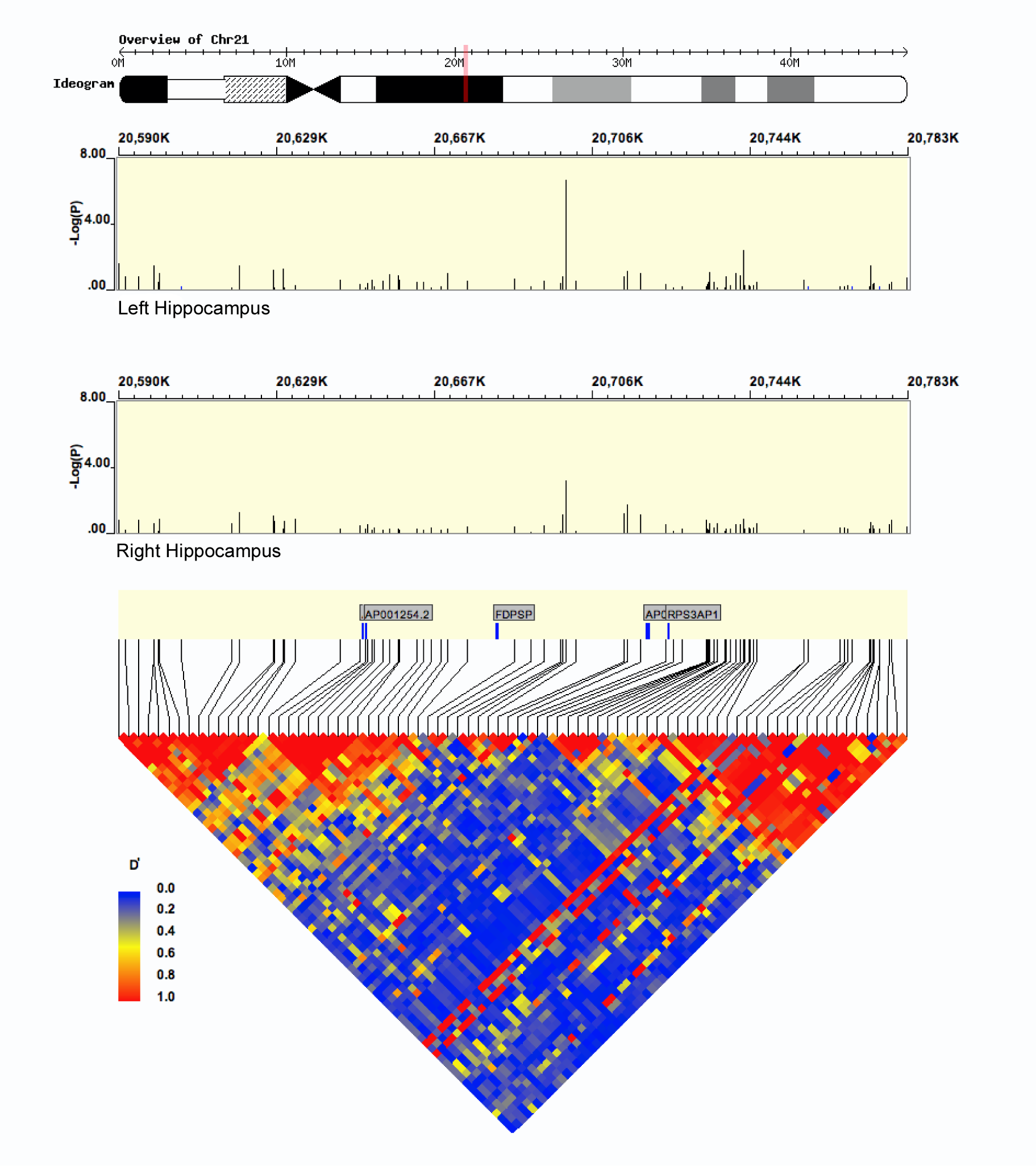

Supplement: Figure S20 — QT analysis of SNPs associated with genes or chromosomal regions as reported in Table 3 of the manuscript. Physical map of the SNPs associated with genes or chromosomal regions in the ADNI sample produced by WGAViewer. The top of the figure is the ideogram of the chromosome; the vertical red line depicts the relative location of locus of interest. Below the graph are the -log p significance values of the individual SNPs on the imaging phenotype (hippocampal atrophy) for the left and right hemispheres as indicated in each figure. The blue lines below the graph indicate the location of the exons in the transcripts annotated (translated region of the DNA). The vertical lines above the accompanying triangular matrix indicate the SNP locations, and demonstrate the LD pattern between SNPs (D'). The warmer colors on the flame scale indicate greater LD while the blue indicates absence of LD. (0.90 MB TIF) [file pone.0006501.s020.tif]
